# Systematic Review of Safety and Efficacy of Rituximab in Treating Immune-Mediated Disorders

**DOI:** 10.3389/fimmu.2019.01990

**Published:** 2019-09-06

**Authors:** Celine Kaegi, Benjamin Wuest, Jens Schreiner, Urs C. Steiner, Alessandra Vultaggio, Andrea Matucci, Catherine Crowley, Onur Boyman

**Affiliations:** ^1^Department of Immunology, University Hospital Zurich, Zurich, Switzerland; ^2^Department of Biomedicine, Azienda Ospedaliero Universitaria Careggi, Florence, Italy; ^3^Faculty of Medicine, University of Zurich, Zurich, Switzerland

**Keywords:** rituximab, CD20, B cell, monoclonal antibody, immune-mediated disease, autoimmune disease, inflammatory disease

## Abstract

**Background:** During the past years biologic agents (also termed biologicals or biologics) have become a crucial treatment option in immunological diseases. Numerous articles have been published on biologicals, which complicates the decision making process on the use of the most appropriate biologic for a given immune-mediated disease. This systematic review is the first of a series of articles assessing the safety and efficacy of B cell-targeting biologics for the treatment of immune-mediated diseases.

**Objective:** To evaluate rituximab's safety and efficacy for the treatment of immune-mediated disorders compared to placebo, conventional treatment, or other biologics.

**Methods:** The PRISMA checklist guided the reporting of the data. We searched the PubMed database between 4 October 2016 and 26 July 2018 concentrating on immune-mediated disorders.

**Results:** The literature search identified 19,665 articles. After screening titles and abstracts against the inclusion and exclusion criteria and assessing full texts, 105 articles were finally included in a narrative synthesis.

**Conclusions:** Rituximab is both safe and effective for the treatment of acquired angioedema with C1-inhibitor deficiency, ANCA-associated vasculitis, autoimmune hemolytic anemia, Behçet's disease, bullous pemphigoid, Castleman's disease, cryoglobulinemia, Goodpasture's disease, IgG4-related disease, immune thrombocytopenia, juvenile idiopathic arthritis, relapsing-remitting multiple sclerosis, myasthenia gravis, nephrotic syndrome, neuromyelitis optica, pemphigus, rheumatoid arthritis, spondyloarthropathy, and systemic sclerosis. Conversely, rituximab failed to show an effect for antiphospholipid syndrome, autoimmune hepatitis, IgA nephropathy, inflammatory myositis, primary-progressive multiple sclerosis, systemic lupus erythematosus, and ulcerative colitis. Finally, mixed results were reported for membranous nephropathy, primary Sjögren's syndrome and Graves' disease, therefore warranting better quality trials with larger patient numbers.

## Introduction

Most immune-mediated disorders are thought to arise due to a deficiency in immune tolerance or an imbalance of immune activation and immune tolerance. Previously, treatment options included corticosteroids and immunosuppressive drugs, which also affect protective immunity and often lead to serious side effects. Since their emergence for the treatment of immunological diseases, the number of biological agents (also termed biologicals or biologics) has grown rapidly and numerous studies assessing their properties have been published. Consequently, to have an overview on the safety, efficacy, and impact biologicals have on quality of life (QoL) is difficult. Therefore, this systematic review is the first of a series of articles focusing on the safety and efficacy of B cell-targeting biologics for the treatment of immune-mediated diseases. The series will concentrate on molecules targeting either B cell-activating factor (BAFF; also known as TNF ligand superfamily member 13B or B lymphocyte stimulator) or CD20. BAFF is produced by myeloid cells and radiation-resistant stromal cells in lymphoid follicles as well as the bone marrow. It plays a major role in the survival and maturation of follicular B cells. The function of the surface molecule CD20 is not quite clear yet. However, monoclonal antibodies directed against CD20 or BAFF affect the B cell population and, therefore, induce a decrease in the production of antibodies.

The aim of this systematic review is to provide an overview on the (i) efficacy and (ii) safety of the anti-human CD20 monoclonal antibody rituximab (RTX) compared to placebo, conventional treatment or other biologics in patients suffering from immune-mediated disorders.

## Methods

### Study Design and Protocol Registration

The PRISMA checklist guided the reporting of this systematic review (Table 1) (1). Our protocol was registered with PROSPERO number CRD42018104726. During the course of our research we found that a considerable number of case series did not state whether they were conducted prospectively or retrospectively. We thus amended this subpoint of our search strategy and included case series with at least three patients when the study design was prospective or unknown, whereas studies stating they were retrospective were excluded.

**Table 1:** The preferred reporting of systematic reviews and meta-analysis (PRISMA) checklist.

**Section/topic**	**#**	**Checklist item**	**Reported on page #**
**TITLE**			
Title	1	Identify the report as a systematic review, meta-analysis, or both	1
**ABSTRACT**			
Structured summary	2	Provide a structured summary including, as applicable: background; objectives; data sources; study eligibility criteria, participants, and interventions; study appraisal and synthesis methods; results; limitations; conclusions and implications of key findings; systematic review registration number	1
**INTRODUCTION**			
Rationale	3	Describe the rationale for the review in the context of what is already known	2
Objectives	4	Provide an explicit statement of questions being addressed with reference to participants, interventions, comparisons, outcomes, and study design (PICOS)	2
**METHODS**			
Protocol and registration	5	Indicate if a review protocol exists, if and where it can be accessed (e.g., Web address), and, if available, provide registration information including registration number	2
Eligibility criteria	6	Specify study characteristics (e.g., PICOS, length of follow-up) and report characteristics (e.g., years considered, language, publication status) used as criteria for eligibility, giving rationale	2
Information sources	7	Describe all information sources (e.g., databases with dates of coverage, contact with study authors to identify additional studies) in the search and date last searched	2
Search	8	Present full electronic search strategy for at least one database, including any limits used, such that it could be repeated	2
Study selection	9	State the process for selecting studies (i.e., screening, eligibility, included in systematic review, and, if applicable, included in the meta-analysis)	2
Data items	11	List and define all variables for which data were sought (e.g., PICOS, funding sources) and any assumptions and simplifications made	2
Risk of bias in individual studies	12	Describe methods used for assessing risk of bias of individual studies (including specification of whether this was done at the study or outcome level), and how this information is to be used in any data synthesis	2
Summary measures	13	State the principal summary measures (e.g., risk ratio, difference in means)	2
Synthesis of results	14	Describe the methods of handling data and combining results of studies, if done, including measures of consistency (e.g., *I*^2^) for each meta-analysis	2
Risk of bias across studies	15	Specify any assessment of risk of bias that may affect the cumulative evidence (e.g., publication bias, selective reporting within studies)	2
Additional analyses	16	Describe methods of additional analyses (e.g., sensitivity or subgroup analyses, meta-regression), if done, indicating which were pre-specified	NA
**RESULTS**			
Study selection	17	Give numbers of studies screened, assessed for eligibility, and included in the review, with reasons for exclusions at each stage, ideally with a flow diagram	2, Figure 1
Study characteristics	18	For each study, present characteristics for which data were extracted (e.g., study size, PICOS, follow-up period) and provide the citations	2, Table S3
Risk of bias within studies	19	Present data on risk of bias of each study and, if available, any outcome level assessment (see item 12)	12, Table 2
Results of individual studies	20	For all outcomes considered (benefits or harms), present, for each study: (a) simple summary data for each intervention group (b) effect estimates and confidence intervals, ideally with a forest plot	4–12
Synthesis of results	21	Present results of each meta-analysis done, including confidence intervals and measures of consistency	NA
Risk of bias across studies	22	Present results of any assessment of risk of bias across studies (see Item 15)	11–12
Additional analysis	23	Give results of additional analyses, if done (e.g., sensitivity or subgroup analyses, meta-regression [see Item 16])	NA
**DISCUSSION**			
Summary of evidence	24	Summarize the main findings including the strength of evidence for each main outcome; consider their relevance to key groups (e.g., healthcare providers, users, and policy makers)	12
Limitations	25	Discuss limitations at study and outcome level (e.g., risk of bias), and at review-level (e.g., incomplete retrieval of identified research, reporting bias)	13
Conclusions	26	Provide a general interpretation of the results in the context of other evidence, and implications for future research	13
**FUNDING**			
Funding	27	Describe sources of funding for the systematic review and other support (e.g., supply of data); role of funders for the systematic review.	15

### Search Strategy

We searched the PubMed database between 4 October 2016 and 26 July 2018. Our full search strategy and research terms were defined in advance (see Table S1). If publications were not available via open or institutional access, study authors were contacted.

### Eligibility Criteria

We included randomized controlled trials (RCTs), their extension trials and their substudies with predefined endpoints. If there were no RCTs, we included prospective case series including at least three patients and non-randomized clinical studies with at least five patients per intervention group. We excluded retrospective trials, *post hoc*-analyses, meta-analyses, reviews, studies made from registries and studies carried out on animal models or where the primary endpoint was non-clinical. Studies had to be available in English or German.

### Study Selection, Data Collection Process, and Analysis

Three authors (CK, BW, and OB) developed and tested a data extraction sheet, whereupon five authors independently (CK, BW, US, AV, and AM) searched PubMed according to the predefined search terms, checked titles and abstracts, carried out a full-text review of the selected studies, and extracted the relevant data. Any disagreements about study inclusion were resolved by consensus.

### Risk of Bias Assessment

CK, JS, and CC used a modified version of the Downs and Black tool (see Table S2) to undertake a quality assessment including a risk of bias of the studies recovered (2). The studies were scored out of a maximum of 28 points for the following categories: (i) reporting, (ii) external validity, (iii) internal validity, and (iv) power, and the scores were summed and ranked high, medium and low quality. Any discrepancies were resolved by consensus.

As we limited our research strategy to the PubMed database, the reference list of these studies, and the expertise of the authors involved, we did not conduct a risk of bias assessment across the studies, as we believed the risk of publication bias would have been high.

### Principal Summary Measures and Synthesis of Results

Our aim was to assess the efficacy and safety of RTX as well as its influence on QoL when used in different immunologic diseases. Since we wanted to give an overview including also rare diseases we did not specify in more detail these endpoints in order not to exclude potentially important studies.

## Results

### Study Selection and Characteristics

**Figure 1:**
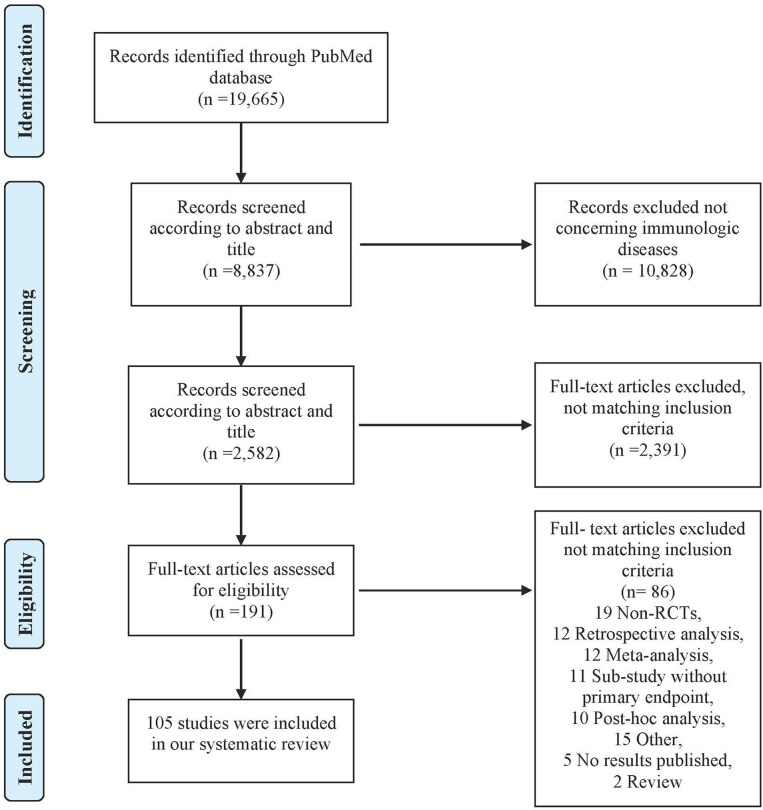
PRISMA diagram of the literature search.

A total of 19,665 articles were identified on PubMed. After screening titles, abstracts and full texts, 105 articles were included in our study (Figure 1). Study characteristics are available in Table S3.

### Synthesized Findings

The following text highlights the most important findings of what is currently known.

#### Acquired Angioedema With C1-Inhibitor Deficiency (C1-INH-AAE)

We found two case series including three patients each, suffering from C1-INH-AAE (3, 4). In both studies, the angioedema attacks were markedly reduced with the use of RTX. Furthermore, most of the patients showed a normalization of C1-inhibitor levels. Health-related QoL was not analyzed. One study did not report any safety events (3). The other study, reported one patient experiencing an adverse event (AE) with no serious adverse event (SAE) (4).

##### Discussion

Available data is derived from unblinded case series of a total of six patients. Further trials with a randomized-controlled design including more patients are needed to verify the available promising results.

#### ANCA-Associated Vasculitis

Of nine trials included, five were randomized and only one of them was double-blind (5–13). The other four trials were either extension trials or substudies. Five hundred and thirty five patients participated in the five original trials, suffering from either granulomatosis with polyangiitis or microscopic polyangiitis. RTX was either used as a maintenance or induction therapy. Its efficacy and safety were compared to azathioprine (AZA), cyclophosphamide (CYC), infliximab, or RTX at a different dosing regimen. All studies allowed concomitant use of corticosteroids. Durations of the different studies ranged from 6 to 28 months and primary endpoints were quite varied.

In the RAVE trial (6) patients were treated with either RTX in combination with CYC or CYC alone. Patients in the CYC group were allowed to switch to AZA. The study failed to reach its primary endpoint, remission of disease with successful prednisone taper by month 6. For all endpoints, RTX treatment was comparable with CYC and AZA. The results remained insignificant during the extension trial (7). However, an open-label extension of this trial showed convincing remission rates after retreatment with RTX and prednisone (8).

The RITUXVAS trial (9) investigated sustained remission as primary endpoint and found no difference between RTX in combination with CYC and CYC alone. Additionally, its extension trial failed to show superiority of RTX when analyzing the composite outcome of death, end-stage renal disease or relapse as primary endpoint (10).

De Menthon et al. (11) compared the efficacy of RTX with infliximab and found numerically but not significantly better results in RTX-treated patients.

The only trial with significant results was the MAINRITSAN study (12), which investigated the use of RTX as maintenance therapy in comparison to AZA. There was a significant reduction in major relapses at month 28, whereas the difference in minor relapses was comparable. The MAINRITSAN2 (13) trial compared a fixed vs. a variable RTX dosing regimen and found no differences concerning the number of relapses at 28 months.

As for safety, studies providing data about AEs showed comparable rates of incidence between RTX and the control groups.

Both the RAVE and the RITUXVAS trial assessed the change in QoL using the 36-item short form health survey (SF-36) score, but did not find any significant improvements (6, 9).

##### Discussion

Since safety assessments were convincing and efficacy measurements were comparable with standard of care treatment, RTX was approved by the Food and Drug Administration (FDA) and the European Medicines Agency (EMA) for the treatment of granulomatosis with polyangiitis and microscopic polyangiitis.

#### Antiphospholipid Syndrome

Only one uncontrolled prospective, open-label trial conducted over a period of 12 months testing efficacy and safety of RTX in 19 patients with antiphospholipid syndrome met our inclusion criteria (14). Patients with a diagnosis of systemic lupus erythematosus (SLE) or systemic autoimmune disease were excluded. Regular therapeutic medication for this disease was allowed.

Assessment of thrombocytopenia, cardiac valve disease, skin ulcers, antiphospholipid nephropathy, and cognitive dysfunction did not reveal a substantial therapeutic effect. Forty nine AEs and 12 SAEs were reported during the 12 month study period. With regard to QoL, there were no significant changes in the SF-36 and patient global assessment (PGA) score at 24 weeks.

##### Discussion

Available data is limited to one uncontrolled prospective, open-label trial and the results of this study in 19 patients did not show any significant therapeutic effect.

#### Autoimmune Hemolytic Anemia

Two RCTs, one double-blind and the other open-label, comprising 96 patients were included in our analysis (15, 16). Only patients with a warm autoimmune hemolytic anemia with a positive direct antiglobulin test were included. Study duration was 1 and 2 years, respectively.

Both trials showed significantly higher response rates in patients receiving additional RTX after 12 months of treatment compared to corticosteroid treatment alone and did not report any significant differences concerning blood cell transfusion, splenectomy, hospitalizations, and corticosteroid dose. While the RAIHA trial (16) provided no information on AEs, Birgens et al. (15) reported no significant difference in the occurrence of AEs between both arms of their study. Neither study analyzed QoL.

##### Discussion

Available studies were conducted as RCTs and showed a marked superiority of RTX in combination with corticosteroids as compared to corticosteroids alone. However, RTX was only tested in 96 patients and further studies conducted in larger groups of patients are needed to confirm these initial findings.

#### Autoimmune Hepatitis

Only one open-label trial assessing six patients conducted over a 72 week period met our inclusion criteria (17). The diagnosis of autoimmune hepatitis had to be proven by biopsy. All included patients had an inadequate repsonse to prednisone and/or AZA.

The primary endpoint was safety with only two patients experiencing AEs, none of them considered serious. After 24 weeks there was a significant change in aspartate aminotransferase (AST) (*p* = 0.032), but not alanine aminotransferase (ALT) (*p* = 0.068), bilirubin, gammaglobulin, and IgG levels. There was no significant change in the nine-point fatigue severity scale.

##### Discussion

Only one unblinded trial without a clinical endpoint matched our inclusion criteria. Further studies are needed to make a suggestion about the efficacy of RTX in autoimmune hepatitis.

#### Behçet's Disease

One randomized-controlled investigator-blinded study analyzing the total adjusted disease activity index (TADAI) as primary endpoint was included (18). The 20 patients had refractory disease with long-standing ocular involvement. The control group received AZA, CYC, and corticosteroid treatment, whereas the RTX group was also given methotrexate (MTX) and prednisolone.

The study showed a significant improvement in TADAI (*p* = 0.009), posterior uveitis and ocular edema, which was however not superior to the comparator (*p* = 0.2). Seven patients from the RTX group reported at least one AE, compared to one AE in the comparator group. QoL was not assessed.

##### Discussion

Provided information is scant and further studies are necessary to analyze RTX in patients with Behçet's disease.

#### Bullous Pemphigoid

Hall et al. published a case series comprising seven patients with bullous pemphigoid conducted over a period of 12 months (19). Patients had persistent disease despite the use of prednisone.

Disease activity was significantly improved with no new skin lesions appearing. This correlated with a significant decrease in anti-BP180 antibody levels. There were no SAEs reported and there was no information about the frequency of AEs.

##### Discussion

Although there is only one case series available, the results are promising. However, RCTs including a sufficient number of patients are needed to prove those findings.

#### Castleman's Disease

We found eight trials eligible for inclusion (20–27). Studies were either case series or open-label trials without a control group. In total 81 patients suffering from multicentric Castleman's disease were treated with RTX. Diagnosis had to be proven by biopsy and patients had to have associated human immunodeficiency virus (HIV) infection, except in one trial (23).

The only trial with a predefined primary endpoint stated that 92% of the patients achieved sustained remission off chemotherapy (22). A great proportion of patients in the other studies also achieved remission. Four trials reported a positive influence on the Kaposi sarcoma-associated herpes virus viral load (21, 24, 26, 27). Although reporting of AEs was incomplete, aggravation of Kaposi sarcoma was a point of concern.

##### Discussion

There are only case series and one open-label study available, however, available results seemed promising. Reactivation of Kaposi sarcoma was an important AE.

#### Cryoglobulinemia

Three unblinded RCTs and one follow-up study met our inclusion criteria totaling 118 patients (28–31). Except for four patients from the study of De Vita et al. (29), all patients were hepatitis C virus positive. Patients with hepatitis B virus or HIV positivity were excluded. Treatment in the control groups varied markedly.

The primary endpoint was met in all three RCTs. Dammacco et al. (28) reported that more patients receiving a combination therapy of pegylated interferon α (Peg-IFN-α) weekly plus ribavirin daily in combination with RTX 375 mg/m^2^ weekly for 4 weeks reached complete response as compared to Peg-IFN-α and ribavirin alone (*p* < 0.05) (28). Following RTX 1,000 mg on days 0 and 14, De Vita et al. reported a significant reduction in global disease activity as measured by the Birmingham vasculitis activity score after 2 months with sustained response until month 24 and a median duration of clinical response of 18 months (29). Sneller et al. (30) stated a significantly higher remission rate in patients receiving RTX.

Information about AEs was sparse among the studies making an interpretation of safety difficult. QoL was not assessed.

##### Discussion

Available information about the efficacy of RTX in patients with cryoglobulinemia is promising.

#### Goodpasture's Disease

Only one case series including three patients matched our inclusion criteria (32). Patients were followed between 33 and 49 months.

In all three patients, anti-glomerular basement membrane antibodies were no longer detectable. Patients two and three were able to discontinue corticosteroid treatment, while patient one remained dialysis dependent. There were no complications reported.

##### Discussion

Since the only available study included three patients, it is not possible to state whether RTX could be a future treatment option in Goodpasture's disease.

#### Graves' Disease

Two double-blind RCTs with 56 patients with euthyroid function suffering from Graves' orbitopathy were identified for inclusion (33, 34). The control groups received either placebo or methylprednisolone.

The study of Salvi et al. (33) found a significant improvement of the clinical activity score between baseline and week 24 and a significant improvement in eye motility. Contrarily, Stan et al. (34) could not report any significant improvement. AEs occurred more often in patients treated with RTX.

##### Discussion

Currently only two RCTs have investigated the use of RTX reporting of contradictory results. Further trials with more patients are needed to clarify the role of RTX in Graves' disease.

#### IgA Nephropathy

Lafayette et al. conducted an open-label RCT with 34 patients suffering from IgA nephropathy and concomitant proteinuria (35). Authors observed no difference between the treatment arms concerning proteinuria or estimated glomerular filtration rate (eGFR), which were the primary endpoints. They reported mild AEs in the RTX group but no SAEs.

##### Discussion

The only available trial found no significant difference between RTX and standard-of-care treatment.

#### IgG4-Related Disease

Five non-randomized trials comprising 52 patients were included. None of the trials included a control group and four were stated as case series. Follow-up ranged from 4 to 48 months.

In the three studies investigating RTX as an induction treatment, RTX led to reduction of IgG4 serum levels and enabled patients to reduce or even discontinue corticosteroids (36–39). Furthermore, Yamamoto et al. (39) reported similar findings when RTX was used in disease relapses. In patients with IgG4-related kidney disease, RTX led to improvement in eGFR and reduction in inflammatory retroperitoneal fibrous tissue in patients with retroperitoneal fibrosis (40).

The incidence of AEs and SAEs were incompletely reported in the studies. Regarding QoL, Carruthers et al. (36) reported a significant decrease in PGA (*p* < 0.00001) with 23/30 patients achieving a PGA score of 0.

##### Discussion

The presented information seems promising in terms of a corticosteroid-sparing effect of RTX, however, no blinded RCTs in IgG4-related disease exist to date.

#### Immune Thrombocytopenia

Five open-label and two double-blind RCTs met the inclusion criteria of our systematic review (41–47). In total 644 patients were treated with RTX or when assigned to the control group either corticosteroids, placebo, vincristine or a RTX, CYC, vincristine, prednisone (R-CVP) regime. Follow-up was between 6 months and 3 years.

Two of the three studies investigating the efficacy of RTX vs. corticosteroids found significantly higher sustained response rates in the RTX group (44, 46, 47). However, the third study found no significant difference (46). In comparison to placebo, RTX led to a reduction in treatment failure, a significantly prolonged time to relapse, and higher platelet counts (41, 43). There was no significant difference in response rates between patients re-treated with RTX and those re-treated with R-CVP (45).

Dai et al. (42) included 50 pediatric patients in their RCT evaluating the efficacy of RTX compared to vincristine. They found that response rates in the RTX group were significantly higher and therefore concluded that standard dose (375 mg/m^2^) RTX is an effective and preferred therapy for children with immune thrombocytopenia, without SAEs.

Most of the studies stated AEs to be of mild or moderate intensity. SAEs were reported by four of the seven studies with one study reporting a significantly increased rate of SAEs. QoL was evaluated in one study using the SF-36 showing no significant changes (41).

##### Discussion

The use of RTX in adult and pediatric patients with ITP showed efficacy while having an acceptable safety profile.

#### Inflammatory Myositis

One double-blind RCT was found investigating the use of RTX in children and adults suffering from either poly- or dermatomyositis (48). Study duration was 44 weeks. Two hundred patients were randomized to either RTX early, receiving RTX at weeks 0 and 1 and placebo at weeks 8 and 9, or the RTX late arm with a reversed treatment regimen. Concomitant use of corticosteroids and immunosuppressive agents was allowed. Results showed no significant difference in time to reach the improvement threshold. As study arms received RTX, AEs were mostly from infusion reactions or infections.

##### Discussion

Available information is derived from one double-blind RCT showing no significant difference concerning the efficacy of RTX.

#### Juvenile Idiopathic Arthritis

Two studies were included, one open-label trial with 55 patients and one case series with three patients (49, 50). All 58 patients suffered from severe active systemic juvenile idiopathic arthritis with an inadequate response to standard treatment. Primary endpoint of the open-label trial was an American College of Rheumatology (ACR) pediatric 30 response at week 24.

The case series by Narvaez et al. (49) demonstrated significant clinical improvement in all three patients. Alexeeva et al. (50) showed a significant improvement in the American College of Rheumatology Pediatric 30 response rate with 98% of the patients reaching it at week 24. Systemic manifestations were significantly reduced by week 12. After 1 year of treatment, 75% of the patients reached clinical remission.

An infusion reaction during the second infusion was the only AE occurring in the case series. There were no SAEs and no deaths reported. However, the open-label trial registered 101 AEs with the most common AE being infusion reactions. There was no information about SAEs and deaths.

QoL was assessed using the childhood health assessment questionnaire (HAQ) score and showed a significant improvement after 12 weeks.

##### Discussion

Currently available results seem very promising and support further investigation in bigger randomized-controlled and double-blind trials.

#### Membranous Nephropathy

Only one open-label RCT on the use of RTX in patients with membranous nephropathy met the inclusion criteria and time frame of our systematic review. In the included RCT, diagnosis of membranous nephropathy had to be proven by biopsy and patients had to have a non-response to non-immunosuppressive antiproteinuric treatment (NIAT). Seventy five patients were treated with either RTX in combination with NIAT or NIAT alone over a period of 6 months (51).

There was no noteworthy difference concerning the primary endpoint, i.e., remission after 6 months. Nonetheless, significantly more patients achieved remission during the follow-up period. There was no information about AEs. However, five patients in the RTX group and four in the NIAT group experienced a SAE.

##### Discussion

The included trial led to contradictory results. A recent RCT became available during the publication of this manuscript and, thus, did not meet the time frame criterion of our systematic review. This recent RCT showed that RTX was equivalent to cyclosporine in inducing remission of proteinuria in patients with membranous nephropathy and superior in maintaining proteinuria remission up to 24 months (52).

#### Multiple Sclerosis

Three double-blind placebo-controlled RCTs totaling 570 patients fit our inclusion criteria (53–55). Study duration ranged from 48 to 122 weeks. Each study was conducted in a different form of multiple sclerosis (MS). Hauser et al. (53) included patients with relapsing-remitting MS (RRMS) with at least one relapse during the past year but no relapse within the last 30 days. Hawker et al. (54) investigated the efficacy of RTX in patients with primary progressive MS (PPMS) without history of relapses, and the RIVITALISE trial only included patients with secondary progressive MS with no relapse during the last year (55). The latter study was the only one using intrathecal RTX. Only Hauser et al. and Hawker et al. had predefined endpoints which were the total count of gadolinium-enhancing lesions on T1-weighted MRI scans and time to confirmed disease progression (53, 54).

One hundred and four patients with RRMS were treated with either RTX or placebo. RTX treated patients experienced a notable drop in the annualized relapse rate and MRI showed a significant reduction in the total number of gadolinium-enhancing lesions (53). Conversely, patients with PPMS failed to show a significant difference in time to confirmed disease progression between RTX and placebo (54). Likewise, RTX did not show efficacy in patients with secondary progressive MS with no relapse during the last year and, thus, the RIVITALISE trial was terminated prematurely and results and AEs were not analyzed (55).

98.6 and 13% of the patients with RRMS treated with RTX and 100 and 14.3% of the patients treated with placebo had at least one AE or SAE, respectively. Patients with PPMS had a 99% incidence of AEs in the RTX group and 100% in the placebo group. SAEs occurred with a frequency of 13.6–16.4%. Two patients treated with RTX and two treated with placebo died.

##### Discussion

Although the sample size was small, results in patients with RRMS seemed very promising and support the initiation of further studies using RTX. In patients with PPMS the outcomes were less convincing. However, available data favors a RTX vs. a placebo treatment. Thus, a treatment benefit cannot be ruled out. Results in patients with secondary progressive MS are not reliable due to early termination.

#### Myasthenia Gravis

Nine case series and one uncontrolled open-label trial with its follow-up were analyzed (56–66). In total 108 patients were treated with RTX. Concomitant treatment varied widely. While some patients had to discontinue immunosuppressive agents, others were allowed to continue. None of the case series had predefined endpoints. The change in manual muscle testing score was the primary endpoint of the open-label trial and its follow-up study.

The only study with a predefined endpoint achieved it showing a significant improvement in the manual muscle testing score as well as a significant reduction in the need for plasma exchange, intravenous immunoglobulin, and prednisone. These results were reflected also during the follow-up trial. Concerning other studies, all of them reported an improvement in disease symptoms. In addition, most patients were able to taper corticosteroids, immunosuppressive treatment, and cholinesterase inhibitors. However, patients with anti-muscle-specific kinase antibodies tended to show better responses than patients with anti-acetylcholine receptor antibodies (57–64, 66).

Only three studies reported on AEs (56, 57, 61). QoL was assessed in one trial using the Myasthenia Gravis-Specific Quality of Life Score 15 but failed to demonstrate any significant improvement (66).

##### Discussion

Although only case series and uncontrolled open-label trials are available, current data are very promising, showing an improvement in disease symptoms and the possibility to taper corticosteroids as well as cholinesterase inhibitors. Further trials investigating the use of RTX in patients with myasthenia gravis are thus warranted.

#### Nephrotic Syndrome

Six clinical trials with idiopathic nephrotic syndrome matching our inclusion criteria were identified, including one follow-up trial (67) and five RCTs (68–72). All but one RCT was conducted open-label (68). Two hundred and eighty three pediatric patients participated in either of the five RCTs. A diagnosis of idiopathic nephrotic syndrome was required in all studies as well as an eGFR of at least 60 ml/min. All patients received concomitant prednisone, which was tapered during the study period. Concomitant use of angiotensin-converting enzyme inhibitors and angiotensin II receptor blockers was allowed.

Only one of the three trials assessing proteinuria as primary endpoint found a significantly better result in RTX-treated patients (67). However, the reported median relapse-free period was significantly longer in RTX patients with a significantly reduced relapse rate (67–69, 71, 72). Additionally, required corticosteroid doses were significantly lower with concomitant RTX. Reliable information about the incidence of AEs, SAEs, and deaths were provided by two studies showing a comparable incidence between RTX, tacrolimus and placebo (68, 72). None of the studies assessed QoL.

##### Discussion

Although RTX treatment did not affect proteinuria, such treatment significantly reduced the relapse rate and the required dose of corticosteroids. Information about safety was lacking.

#### Neuromyelitis Optica

We found one open-label RCT in patients with neuromyelitis optica (73). It included 86 patients with a neuromyelitis optica spectrum disorder and an Expanded Disability Status Scale of 0–7 in the previous 12 months. The control group received AZA in combination with prednisolone which was tapered over time.

The primary endpoint was the annual relapse rate after 12 months, which was achieved. Furthermore, RTX led to a significant decrease in the Expanded Disability Status Scale when compared to AZA. The incidence of AEs was not significantly different.

##### Discussion

In the only available RCT, RTX proved its superiority over AZA in the treatment of neuromyelitis optica. However, further blinded studies are needed to confirm this.

#### Pemphigus

Two RCTs matched our inclusion criteria (74, 75). One of them was observer blinded, the other was an open-label trial. One hundred and twelve patients with either pemphigus vulgaris or pemphigus foliaceus were treated with RTX or prednisone.

Kanwar et al. (74) assessed the efficacy of different RTX dosing regimens and concluded that there was no difference in the time taken to achieve any of the primary endpoints (partial and complete remission). However, relapse seemed to be more common in patients who received low-dose RTX (74). Joly et al. (75) demonstrated that significantly more patients treated with a combination of RTX and a corticosteroid achieved remission off corticosteroids after 24 months as compared to patients treated with corticosteroids solely. Furthermore, these patients achieved remission significantly faster and time of remission off corticosteroids was significantly longer.

The dermatology life quality index and skindex scores were significantly better in patients treated with RTX, SAEs, and AEs were not significantly different between the groups.

##### Discussion

RTX proved its steroid-sparing effect and furthermore led to a significantly longer remission duration off corticosteroids. The FDA and EMA approved its use for moderate-to-severe pemphigus vulgaris in adult patients.

#### Rheumatoid Arthritis

Nineteen trials matching our inclusion criteria were identified (76–94). All but five studies were double-blind. RTX was compared to placebo, MTX, leflunomide, TNF inhibitors, or itself at different dosages. Major inclusion criteria was a diagnosis of rheumatoid arthritis (RA) usually according to the ACR criteria. Except for one trial, patients needed to suffer from active disease. The most frequently stated exclusion criteria were a concomitant autoimmune disease other than RA and a systemic involvement of RA.

Ten trials including one follow-up trial investigated the use of RTX in comparison to placebo or disease-modifying antirheumatic drugs (DMARDs) in patients with active RA despite the use of MTX (77–79, 83–86, 88, 92, 94). In most of the studies, a combination therapy of RTX and MTX was superior to placebo and MTX in terms of efficacy. In addition, Edwards et al. (77) reported significantly better results when RTX plus MTX or RTX plus CYC was used in comparison to MTX monotherapy. However, RTX monotherapy was not significantly better than MTX monotherapy when analyzing ACR response rates. Owczarczyk et al. (83) did not predefine an endpoint, and therefore failed to show significant differences between RTX and MTX monotherapy concerning the disease activity score (DAS) 28 and EULAR (European League Against Rheumatism) response. Peterfy et al. (84) investigated the change in the RA magnetic resonance imaging erosion score and found a significant reduction in radiographic progression at week 24 in RTX-treated patients irrespective of the dosage used as compared to placebo.

The REFLEX trial was conducted in patients with active RA despite the use of one or more TNF inhibitors and investigated the ACR20 response rate at 24 weeks as its primary endpoint (76). It also found RTX in combination with MTX to be significantly superior to MTX monotherapy. A substudy of this trial observed superiority of RTX combination therapy in the Genant-modified Sharp score at week 56 (90). Similar results were reported by the SUNRISE trial assessing the ACR20 response rate at week 48 (82).

SMART, a further trial conducted in TNF inhibitor-resistant patients tested a retreatment after 24 weeks with either one or two doses of 1,000 mg RTX and found no statistically significant difference between these dosing regimens (81).

The DANCER, SERENE, and MIRROR trials aimed to assess the effectiveness of different doses of RTX (500 mg vs. 1,000 mg) in combination with MTX. In these trials, RTX-treated patients achieved significantly higher ACR20 response rates as compared to placebo, independent of the dose administered (78, 79, 92). Furthermore, there was no significant difference between the two dosing regimens as reported by the DANCER and MIRROR trials concerning the ACR50/70 response rates.

Vital et al. (94) compared an induction therapy of RTX given on weeks 0, 2, and 4, to an induction therapy with only two doses given on weeks 0 and 2. They found a significantly greater B cell depletion paralleled by significantly better EULAR and ACR20 response rates at 40 and 52 weeks in patients with an induction treatment of three doses. Furthermore, they demonstrated that immunoglobulin titers remained stable in both arms, and AEs were balanced.

When assessing RTX plus a TNF inhibitor and MTX, Greenwald et al. (80) concluded that the preliminary safety profile of RTX plus a TNF inhibitor and MTX was consistent with the safety profile of RTX plus MTX without a TNF inhibitor, with no new safety signals observed. However, in comparison to the control group, receiving placebo plus MTX and a TNF inhibitor, AEs and SAEs were numerically more frequent in the RTX group, and there was no clear evidence of an efficacy advantage in patients receiving RTX in combination with a TNF inhibitor and MTX.

Some investigations have turned their attention to trying to find valid alternatives to RTX. Wijesinghe et al. (86) assessed ACR20/50/70 response rates and found no significant difference between patients treated with RTX and those treated with leflunomide.

A recent trial published compared abatacept, an alternative TNF inhibitor and RTX in patients with an inadequate response to TNF inhibitors and MTX, and found no significant difference concerning DAS28 response at 24 weeks, which was the primary endpoint (87). Yet, abatacept tended to be less effective when analyzing ACR20, EULAR response, the clinical disease activity index and simple disease activity index. However, there were too few patients included to draw a clear conclusion.

Since study duration, premedication, dosing regimen, and control group treatment varied markedly, a comparison of different incidence rates of AEs is difficult. Five trials did not report about AEs at all (83, 86, 88, 90, 91). None of the trials analyzing safety reported a significantly increased rate of AEs, SAEs, or deaths.

Various trials assessed QoL. Seven reported a significant improvement in HAQ disability index (76–79, 89, 90, 92, 93), four in functional assessment of chronic illness therapy—fatigue scale (76, 78, 79, 92), and three in SF-36 (76, 78, 92). The SUNRISE, ORBIT, SMART, and SWITCH trials as well as the study of Wijesinghe et al. (86) found no significant improvement in QoL measures (82, 85, 87).

##### Discussion

The use of RTX in patients with RA was approved by the FDA and the EMA. However, its use is restricted to patients suffering from severe (EMA) or moderate-to-severe (FDA) active disease with an inadequate response to previous treatment options of DMARDs including at least one TNF inhibitor. RTX has to be administered in combination with MTX and an appropriate premedication to prevent infusion reactions. Newer studies aim to prove the non-inferiority of biosimilars to RTX.

#### Sjögren's Syndrome

Four double-blind RCTs and one substudy were identified (95–99). All 298 included patients had a diagnosis of primary Sjögren's syndrome according to the American-European Consensus Group Criteria for primary Sjögren's syndrome. All studies compared RTX to placebo.

Three out of five studies failed to achieve their primary endpoint (95, 96, 98). The study of Dass et al. (95) could not show a superiority of RTX concerning a 20% improvement in a visual analog scale to evaluate fatigue severity (VAS-F). Neither did the study of Bowman et al. (98) assessing a 30% reduction in VAS-F or oral dryness. The TEARS trial did not meet its primary endpoint either (96). However, significantly more patients treated with RTX reached the primary endpoint in the study of Meijer et al. (97) with a significant improvement in the stimulated whole saliva flow rate.

Three trials evaluated the influence of RTX treatment on QoL using the SF-36 score. While two studies found a significant improvement (95, 97), the third failed to show any difference (98).

The TEARS trial and the study of Bowman et al. were the only studies providing reliable data on safety events. Despite the use of acetaminophen and corticosteroid as premedication, infusion reactions occurred significantly more often in the RTX group (96). Other AEs were comparable.

##### Discussion

Although three out of five trials did not meet their primary endpoint, RTX may be a possible treatment option for patients suffering from primary Sjögren syndrome. Thus, further trials with an appropriate dosing schedule including more patients are needed to draw a clear conclusion.

#### Spondyloarthropathy

Two open-label clinical trials as well as one follow-up trial were included for analysis (100–102). All 29 patients were treated with RTX without a matching control group. One study was conducted in patients with active ankylosing spondylitis despite the use of at least two NSAIDs, the other comprised patients with active psoriatic arthritis despite the use of MTX.

Forty percentage of all patients with ankylosing spondylitis achieved an Assessment of SpondyloArthritis International Society 20 response at week 24, which was the primary endpoint. Remarkably, TNF inhibitor-naïve patients had better treatment outcomes concerning Assessment of SpondyloArthritis International Society 20/40 response, partial remission, and Bath Ankylosing Spondylitis Disease Activity Index 20/50.

Primary endpoint in psoriatic arthritis patients was a psoriatic arthritis response criteria improvement by 30% of tender and swollen joint count or if only one fulfilled, an additional 30% improvement of patient or evaluator global assessment. It was met by 56% of all patients. However, dactylitis remained stable, while enthesitis symptoms significantly improved.

Safety reporting was incomplete with only two studies reporting AEs (100, 101). QoL was only analyzed in patients with psoriatic arthritis and showed a significant improvement.

##### Discussion

Results in ankylosing spondylitis and psoriatic arthritis patients were promising with improvements in Assessment of SpondyloArthritis International Society response, psoriatic arthritis response criteria and DAS28. Nevertheless, both trials were conducted without a control group making an interpretation of RTX's efficacy difficult. Furthermore, safety reporting was poor.

#### Systemic Lupus Erythematosus

We found four RCTs matching our inclusion criteria (103–106), with three of them conducted in a double-blind manner. Five hundred and four patients with a diagnosis of SLE according to the ACR criteria were randomized. Three studies were conducted in patients with concomitant lupus nephritis. Patients were randomized to either RTX, CYC, or placebo.

Zhang et al. (105) compared RTX in combination with CYC to CYC alone and found a significantly higher response rate in terms of the amount of proteinuria during 1 day and a higher serum albumin concentration in patients receiving a combination of RTX and CYC. The LUNAR trial, comparing the efficacy of RTX to placebo in lupus nephritis patients, found no significant differences concerning the primary endpoints (104). The EXPLORER study using RTX in SLE patients found also no indication for a superiority of RTX above placebo concerning clinical response rate. However, a subanalysis found significantly better results in African American and Hispanic SLE patients (103).

The LUNAR trial was the only study reporting AE frequency with 98.6% of RTX-treated patients and 95.8% of placebo-treated patients experiencing at least one AE (104). SAEs and deaths were reported by three of the studies and showed similar incidence rates of RTX and placebo (103, 104). The two studies investigating QoL using the SF-36 score found no significant improvement (103, 104).

##### Discussion

Efficacy of RTX was assessed using a definition of complete response, major clinical response, and renal response. Only one trial showed a superiority of RTX over a conventional treatment (105). However, two substudies came to the conclusion that African American and Hispanic SLE patients may profit from a treatment. Thus, further studies investigating this supposition are necessary.

#### Systemic Sclerosis

One open-label trial with blinded outcome assessors including 14 patients met our inclusion criteria (107). A diagnosis of diffuse systemic sclerosis according to the ACR criteria was the main inclusion criteria. Furthermore, patients needed to be anti-Scl-70 autoantibody positive and had to have an interstitial lung disease. The trial compared efficacy of RTX vs. standard-of-care treatment.

Patients treated with RTX showed a significant improvement in forced vital capacity, diffusing capacity of the lungs for carbon monoxide (DLCO) and modified Rodnan skin score after 1 year, while patients receiving standard of care showed a deterioration in forced vital capacity and DLCO.

Safety outcomes were poorly reported. The assessment of QoL showed a significant improvement after 1 year of RTX treatment using the HAQ score.

##### Discussion

The only available trial showed promising results concerning lung function and skin thickening. Nevertheless, the trial was rather small and unblinded, and safety outcomes were poorly reported.

#### Ulcerative Colitis

One double-blind RCT conducted over a 24 week time period was eligible for our review (108). Twenty four patients fulfilled the inclusion criteria of moderately active ulcerative colitis with inadequate response to corticosteroid treatment. The control group received matching placebo.

The primary endpoint, remission after 4 weeks, was not met. Safety was comparable between RTX and placebo. There was no difference concerning the QoL as measured by the inflammatory bowel disease questionnaire.

##### Discussion

RTX treatment in patients with ulcerative colitis did not meet any of the predefined endpoints. Safety was comparable between RTX and placebo.

### Risk of Bias

Results of the risk of bias and quality assessment of individual studies are available in Table 2. Due to the heterogeneous results reporting the primary and secondary outcomes of the trials in individual diseases as well as the high possibility of publication bias we did not carry out a risk of bias assessment across studies.

**Table 2:** Downs and black assessment.

**Study**	**Total Scores**	**Risk of bias**
Stone et al. (6) (RAVE)	20	Medium
Specks et al. (7) (extension of RAVE)	18	Medium
Miloslavsky et al. (8) (open-label extension of RAVE)	13	High
Jones et al. (9) (RITUXVAS)	20	Medium
Jones et al. (10) (extension of RITUXVAS)	17	Medium
Berden et al. (5) (substudy of RITUXVAS)	15	Medium
De Menthon et al. (11)	14	High
Guillevin et al. (12) (MAINRITSAN)	18	Medium
Charles et al. (13) (MAINRITSAN2)	20	Medium
Erkan et al. (14) (RITAPS)	18	Medium
Birgens et al. (15)	22	Low
Michel et al. (16) (RAIHA)	23	Low
Burak et al. (17)	14	High
Davatchi et al. (18)	16	Medium
Hall et al. (19)	13	High
Levi et al. (4)	11	High
Busse et al. (3)	10	High
Marcelin et al. (24)	18	Medium
Ide et al. (23)	17	Medium
Bower et al. (21)	19	Medium
Gerard et al. (22)	18	Medium
Powles et al. (26)	12	High
Bestawros et al. (20)	12	High
Peker et al. (25)	4	High
Uldrick et al. (27)	17	Medium
Dammacco et al. (28)	26	Low
De Vita et al. (29)	21	Low
Quartuccio et al. (31) (Follow-up of De Vita et al.)	16	Medium
Sneller et al. (30)	28	Low
Oddis et al. (48) (RIM trial)	25	Low
Ravani et al. (70)	25	Low
Ravani et al. (67)	17	Medium
Magnasco et al. (69)	24	Low
Iijima et al. (68)	27	Low
Ravani et al. (71)	23	Low
Basu et al. (72) (RITURNS)	26	Low
Dahan et al. (51)	23	Low
Lafayette et al. (35)	23	Low
Shah et al. (32)	5	High
Salvi et al. (33)	21	Low
Stan et al. (34)	24	Low
Khosroshahi et al. (37)	8	High
Khosroshahi et al. (38)	16	Medium
Carruthers et al. (36)	17	Medium
Yamamoto et al. (39)	8	High
Quattrocchio et al. (40)	12	High
Hasan et al. (45) (only considering Part 2)	5	High
Zaja et al. (47)	20	Medium
Li et al. (46)	23	Low
Arnold et al. (41)	27	Low
Gudbrandsdottir et al. (44)	21	Low
Dai et al. (42)	11	High
Ghanima et al. (43) (RITP trial)	25	Low
Narvaez et al. (49)	6	High
Alexeeva et al. (50)	16	Medium
Merrill et al. (103) (EXPLORER trial)	27	Low
Rovin et al. (104) (LUNAR trial)	23	Low
Zhang et al. (105)	10	High
Andrade-Ortega et al. (106)	19	Medium
Hauser et al. (53)	21	Low
Hawker et al. (54)	27	Low
Komori et al. (55) (RIVITALISE trial)	21	Low
Illa et al. (58)	14	High
Lebrun et al. (60)	12	High
Nelson et al. (62)	5	High
Stieglbauer et al. (63)	7	High
Lindberg et al. (61)	9	High
Diaz-Manera et al. (57)	16	Medium
Sun et al. (64)	14	High
Anderson et al. (56)	16	Medium
Beecher et al. (65) (extension trial of Anderson et al.)	13	High
Lebrun et al. (59)	17	Medium
Jing et al. (66)	19	Medium
Nikoo et al. (73)	23	Low
Joly et al. (75) (Ritux 3)	25	Low
Song et al. (101)	17	Medium
Song et al. (102) [Follow-up trial of Song et al. (101)]	15	Medium
Jimenez-Boj et al. (100)	15	Medium
Daoussis et al. (107)	21	Low
Leiper et al. (108)	25	Low
Dass et al. (95)	22	Low
Meijer et al. (97)	27	Low
Devauchelle-Pensec et al. (96) (TEARS trial)	26	Low
Bowman et al. (98) (TRACTISS)	26	Low
Fisher et al. (99) (substudy of TRACTISS)	25	Low
Edwards et al. (77)	26	Low
Strand et al. (88) [2 year follow-up of Edwards et al. (77)]	22	Low
Cohen et al. (76) (REFLEX trial)	26	Low
Keystone et al. (90) (substudy of the REFLEX trial)	26	Low
Emery et al. (79) (DANCER trial)	25	Low
Owczarczyk et al. (83)	12	High
Emery et al. (78) (SERENE trial)	24	Low
Mease et al. (82) (SUNRISE trial)	26	Low
Greenwald et al. (80) (TAME trial)	22	Low
Tak et al. (93) (IMAGE trial)	25	Low
Tak et al. (89) (Extension of the IMAGE trial)	26	Low
Mariette et al. (81) (SMART trial)	22	Low
Vital et al. (94)	23	Low
Peterfy et al. (84) (RA-SCORE trial)	25	Low
Porter et al. (85) (ORBIT trial)	24	Low
Wijesinghe et al. (86)	23	Low
Brown et al. (87) (SWITCH RCT)	22	Low
Rubbert-Roth et al. (92)	28	Low
Bingham et al. (91)	14	High
Kanwar et al. (74)	20	Medium

## Discussion

### Summary of Main Findings

As summarized in Table 3, RTX has been approved by the FDA and EMA for the treatment of ANCA-associated vasculitis, RA and pemphigus vulgaris. RCTs have demonstrated the efficacy and safety of RTX in autoimmune hemolytic anemia, Behçet's disease, cryoglobulinemia, ITP, MS, neuromyelitis optica, and systemic sclerosis. Conversely, results with the use of RTX in patients with inflammatory myositis, primary Sjögren's syndrome, SLE, Grave's disease, and ulcerative colitis were rather negative.

**Table 3:** Summary of the results.

**Disease**	**[5pt]ANCA-associated vasculitis**	**[5pt]Antiphospholipid syndrome**	**[5pt]Autoimmune hemolytic anemia**	**[5pt]Autoimmune hepatitis**	**[5pt]Behçet's disease**	**[5pt]Bullous pemphigoid**	[5pt]C1-INH-AAE^*^	**[5pt]Castleman's disease**	**[5pt]Cryoglobulinemia**	**[5pt]Goodpasture's disease**	**[5pt]Graves' disease**	**[5pt]IgA nephropathy**	**[5pt]IgG4-related disease**	**[5pt]Immune thrombocytopenia**	**[5pt]Inflammatory myositis**	**[5pt]Juvenile idiopathic arthritis**	**[5pt]Membraneous nephropathy**	**[5pt]Multiplesclerosis**		**[5pt]Myasthenia gravis**	**[5pt]Nephrotic syndrome**	**[5pt]Neuromyelitis optica**	**[5pt]Pemphigus**	**[5pt]Rheumathoid arthritis**	**[5pt]Sjörgen's syndrome**	**[5pt]Spondyloarthropathy**	**[5pt]Systemic lupus erythematosus**	**[5pt]Systemic sclerosis**	**[5pt]Ulcerative colitis**
																		**PPMS**	**RRMS**										
Level I																													
Level IIa																													
Level IIb																													
Level IIIa																													
Level IIIb																													
Level IV																													
Too little information																													
**Legend**																													
Level I	Approved by FDA/EMA																												
Level IIa	Multicentric double-blind RCTs proving a significant superiority over standard-of-care treatment																												
Level IIb	Multicentric double-blind RCTs proving a significant superiority over placebo																												
Level IIIa	Clinical study, not fulfilling the above-mentioned criteria, but proving a superiority over standard-of-care treatment																												
Level IIIb	Clinical study, not fulfilling the above mentioned-criteria, but proving a superiority over placebo																												
Level IV	Case series or open-label trials without control group with positive results																												
Achieved																													
Failed																													
Mixed result																													

* *Acquired angioedema with C1-inhibitor deficiency*.

Clinical studies showed promising results in patients with bullous pemphigoid, C1-INH-AAE, Castleman's disease, idiopathic nephrotic syndrome, Goodpasture's syndrome, IgG4-related disease, juvenile idiopathic arthritis, myasthenia gravis, and spondyloarthropathy. Available results for the treatment of IgA nephropathy showed no effects.

Notably, a recent RCT in patients with membranous nephropathy was released during the publication of this manuscript and, thus, could not be included in this systematic review. This RCT showed similar or superior efficacy of RTX in terms of inducing and maintaining reduced proteinuria in patients with membranous nephropathy (52). A more detailed overview of the safety of RTX in individual diseases is given in Table 4.

**Table 4:** Adverse events.

**Organ systems affected**	**Adverse event(s)**		**References**
Systemic	(a) Immediate-type adverse reactions	Cytokine release syndrome, infusion reactions (often present during first RTX infusion and decrease with subsequent infusions)	(9, 12, 14, 18, 22, 27, 30, 33, 35, 38, 41, 44–46, 48–50, 52–54, 56, 57, 67–74, 76, 78–85, 87, 89, 91–93, 95–98, 101, 103, 104, 108)
	(b) Late-type immune mediated adverse reactions	Serum sickness	(95, 97, 101, 103)
	(c) Infection	Most commonly affecting ears, nose and upper respiratory tract; rarely prostatitis, urinary infection, septicaemia, colitis, pyelonephritis and others	(6–8, 12–14, 16, 18, 22, 29–31, 35, 36, 40, 41, 43, 44, 48, 50, 52, 54, 67, 72, 74–87, 89, 92–94, 97, 98, 100, 107, 108)
	(d) Malignancy	Worsening or reactivation of Kaposi sarcoma (in Castleman's disease) Single cases of other malignancies were reported in several studies in study groups involving or not involving RTX treatment, however a connection to RTX treatment was never obvious. For this matter we refer to two systematic reviews, one finding no association of malignancy with RTX (109), the other reporting a slightly increased risk of cutaneous melanoma (110).	(20–22, 24)
Cardiovascular	Venous thrombotic events, pulmonary embolism, cardiovascular such as: myocardial infarction, heart failure, supraventricular tachycardia, dysrhythmia, hypotension, cerebral infarction, angina, coronary artery disease		(6, 7, 9, 12–14, 46–48, 51–53, 77–80, 86, 91, 93)
Gastrointestinal and hepatic	Gastrointestinal symptoms: nausea, reflux disease, stomatitis, anorexia, gastrointestinal bleeding, abdominal pain, diarrhea, appendicitis, increased transaminases, gastritis, pharyngolaryngeal pain, acute liver failure (single case in hepatitis C-related cryoglobulinemia) Reactivation of hepatitis B^***^		(14, 27, 29, 34, 35, 38, 43, 48, 51, 52, 69, 71, 72, 74, 78, 81, 85, 87, 95, 98) (111)
Hematologic events	Cytopenias, including anemia, thrombocytopenia, leukopenia, neutropenia, hypogammaglobulinemia [27%; (112)], and agranulocytosis		(6, 7, 9, 12, 16, 22, 27, 30, 31, 44, 48, 50, 61, 67, 68, 72, 84, 85, 87, 103, 104)
Musculoskeletal	Myalgias, arthralgias, arthritis, rarely bone fractures		(4, 14, 34, 41, 52, 67, 70, 71, 75, 84, 85, 87, 91, 98)
Nervous system (including eyes)	Neuropsychiatric problems, neurologic symptoms such as headache and dizziness, optic neuropathy in Grave's disease, fatigue		(14, 34, 36, 41, 52, 79, 84, 85)
Renal	Renal failure (described in ANCA-associated vasculitis)		(9)
Upper and lower airways	Hemorrhagic alveolitis (single case), pneumonia, bronchospasm, mild dyspnea, throat irritation		(29, 47, 54, 61, 67, 69, 70, 72, 77, 82, 85, 96, 100, 108)
Skin	Rash, itching, pruritus, acneiform eruptions, erythema, purpura		(14, 34, 42, 43, 48, 53, 54, 67, 69–71, 74, 77, 84, 85, 87)

*List of adverse side events per organ system*.

*** *Although these adverse events were not significant in our systematic review, narrative reviews have highlighted these adverse events*.

### Limitations

To our knowledge this is the first synthesis of data on RTX in immune-mediated diseases. We have used standardized systematic overview techniques, which have helped to minimize the risk of bias. Furthermore, we assessed the quality and bias of each study using the Downs and Black checklist (see Table S4).

However, our systematic review has some limitations. Firstly, we included studies with different patient ages, concomitant treatments, premedications, control groups, and study durations making a direct comparison difficult. Secondly, published studies used different primary endpoints, inclusion criteria and dosing regimens making a direct comparison in a meta-analysis very difficult.

### Conclusions

Since RTX first came on the market over 20 years ago, it has offered a new dimension of targeted treatment in many immune-mediated diseases that were previously not or only insufficiently amenable to treatment. A systematic and comprehensive document establishing the safety and efficacy of RTX in different immune-mediated diseases to our knowledge has not yet been published, making our work novel in this field. We narrate the potential use for RTX, demonstrating its safety and efficacy that has led to regulatory approval in certain pathologies and its potential as a valid alternative to others. However, where the safety and efficacy of RTX cannot be established, due to the lack of unbiased trials, we call upon the scientific community to undertake robust RCTs to assess its potential.

## Author Contributions

CK and OB: conception and design of the work. CK, BW, JS, US, AV, AM, and CC: data collection. CK, BW, and CC: data analysis and interpretation. CK, CC, and OB: drafting the article. CK, BW, JS, US, AV, AM, CC, and OB: critical revision of the article and final approval of the version to be published.

### Conflict of Interest Statement

The authors declare that the research was conducted in the absence of any commercial or financial relationships that could be construed as a potential conflict of interest.

## Abbreviations

ACR: American College of Rheumatology
ALT: alanine aminotransferase
AST: aspartate aminotransferase
AZA: azathioprine
CAS: clinical activity score
CYC: cyclophosphamide
C1-INH-AAE: acquired angioedema with C1-inhibitor deficiency
DAS: disease activity score
DLCO: diffusing capacity of the lungs for carbon monoxide
DMARD: disease-modifying antirheumatic drug
eGFR: estimated glomerular filtration rate
EMA: European Medicines Agency
EULAR: European League Against Rheumatism
FDA: United States Food and Drug Administration
HAQ: health assessment questionnaire
HIV: human immunodeficiency virus
ITP: immune thrombocytopenia
MS: multiple sclerosis
MTX: methotrexate
NIAT: non-immunosuppressive antiproteinuric treatment
Peg-IFN-α: pegylated interferon α
PGA: patient global assessment
PPMS: primary progressive MS
QoL: quality of life
RA: rheumatoid arthritis
RCT: randomized controlled trial
R-CVP, RTX, CYC: vincristine and prednisone
RRMS: relapsing-remitting MS
RTX: rituximab
SAE: serious adverse event
SF-36: 36-item short form health survey
TADAI: total adjusted disease activity index
VAS-F: visual analog scale to evaluate fatigue severity.
