# Supplementary material for: Systematic Review of Safety and Efficacy of Rituximab in Treating Immune-Mediated Disorders
Source: Front Immunol. 2019 Sep 6;10:1990. doi: 10.3389/fimmu.2019.01990 (PMC6743223; doi:10.3389/fimmu.2019.01990)
Supplement: Supplementary file 1 [file Table_1.DOCX]

**Table S1. Search terms**

| 1. rituximab AND ANCA associated vasculitis 2. rituximab AND ANCA associated vasculitis Filters: Randomized Controlled Trial |
| --- |
| 1. rituximab AND antiphospholipid syndrome 2. rituximab AND antiphospholipid syndrome Filters: Randomized Controlled Trails |
| 1. rituximab AND autoimmune haemolytic anemia 2. rituximab AND autoimmune haemolytic anemia Filters: Randomized Controlled Trial |
| 1. rituximab AND autoimmune hepatitis 2. rituximab AND autoimmune hepatitis Filters: Randomized Controlled Trial |
| 1. rituximab AND Behcet’s disease 2. rituximab AND Behcet’s disease Filters: Randomized Controlled Trial |
| 1. rituximab AND bullous pemphigoid 2. rituximab AND bullous pemphigoid Filters: Randomized Controlled Trial |
| 1. rituximab AND C1 inhibitor deficiency |
| 1. rituximab AND castlemans disease 2. rituximab AND castlemans disease Filters: Randomized Controlled Trial |
| 1. rituximab AND crohn |
| 1. rituximab AND cryoglobulinemia 2. rituximab AND cryoglobulinemia Filters: Randomized Controlled Trial |
| 1. rituximab AND dermatomyositis 2. rituximab AND dermatomyositis Filters: Randomized Controlled Trial |
| 1. rituximab AND anti-glomerular basement membrane disease 2. rituximab AND goodpasture |
| 1. rituximab AND glomerulonephritis 2. rituximab AND glomerulonephritis Filters: Randomized Controlled Trial |
| 1. rituximab AND graves disease 2. rituximab AND graves disease |
| 1. rituximab AND IgA nephropathy 2. rituximab AND IgA nephropathy Filters: Randomized Controlled Trial |
| 1. rituximab AND IgG4 related disease 2. rituximab AND IgG4 related disease Filters: Randomized Controlled Trial |
| 1. rituximab AND immune thrombocytopenia 2. rituximab AND immune thrombocytopenia Filters: Randomized Controlled Trial |
| 1. rituximab AND juvenile idiopathic arthritis 2. rituximab AND juvenile idiopathic arthritis Filters: Randomized Controlled Trial 3. rituximab AND juvenile arthritis 4. rituximab AND rheumatoid arthritis AND child 5. rituximab AND arthritis AND child |
| 1. rituximab AND lupus 2. rituximab AND lupus Filters: Randomized Controlled Trial 3. rituximab AND SLE 4. rituximab AND SLE Filters: Randomized Controlled Trial |
| 1. rituximab AND myasthenia gravis 2. rituximab AND myasthenia gravis Filters: Randomized Controlled Trial |
| 1. rituximab AND neuromyelitis optica 2. rituximab AND neuromyelitis optica Filters: Randomized Controlled Trial |
| 1. rituximab AND pemphigus vulgaris 2. rituximab AND pemphigus vulgaris Filters: Randomized Controlled Trial |
| 1. rituximab AND PIGN 2. rituximab AND post-infectious nephritis 3. rituximab AND post-infectious glomerulonephritis |
| 1. rituximab AND polymyositis 2. rituximab AND polymyositis Filters: Randomized Controlled Trial |
| 1. rituximab AND psoriasis 2. rituximab AND psoriasis Filters: Randomized Controlled Trial |
| 1. rituximab AND rheumatoid arthritis 2. rituximab AND rheumatoid arthritis Filters: Randomized Controlled Trial |
| 1. rituximab AND sjögrens syndrome 2. rituximab AND sjögrens syndrome Filters: Randomized Controlled Trial |
| 1. rituximab AND spondyloarthropathy 2. rituximab AND spondyloarthropathy Filters: Randomized Controlled Trial |
| 1. rituximab AND systemic sclerosis 2. rituximab AND systemic sclerosis Filters: Randomized Controlled Trial |
| 1. rituximab AND ulcerative colitis |
| 1. rituximab AND uveitis 2. rituximab AND uveitis Filters: Randomized Controlled Trial |
