# Supplementary material for: Systematic Review of Safety and Efficacy of Rituximab in Treating Immune-Mediated Disorders
Source: Front Immunol. 2019 Sep 6;10:1990. doi: 10.3389/fimmu.2019.01990 (PMC6743223; doi:10.3389/fimmu.2019.01990)
Supplement: Supplementary file 3 [file Table_3.DOCX]

| **Table S3. Main characteristics of included studies** | | | | | | | | | | | |
| --- | --- | --- | --- | --- | --- | --- | --- | --- | --- | --- | --- |
| **Acquired angioedema with C1-inhibitor deficiency** | | | | | | | | | | | |
| **Source** | **Patients treated** | **RTX** | **Primary endpoint (1°) investigated** | **Secondary endpoint (2°) investigated** | **Verum reaching 1°** | **Control reaching 1°** | **Verum reaching 2°** | **Control reaching 2°** | **Adverse Events** | **QoL** | **Serious adverts events** |
| Levi et al., 2006 | 3 | 375mg/m^2^ weekly for 4 weeks. | No primary endpoint defined. | No secondary endpoints defined. | No information. | No control. | No information. | No control. | 1 patient with mild diffuse muscle and joint pain | No information. | No SAE |
| Busse et al., 2017 | 3 | 375mg/m^2^ weekly for four weeks (=one cycle). Repeated for various times. | No primary endpoint defined. | No secondary endpoints defined. | No information. | No control. | No information. | No control. | No information. | No information. | No information. |
| **ANCA- associated vasculitis** | | | | | | | | | | | |
| **Source** | **Patients treated** | **RTX** | **Primary endpoint (1°) investigated** | **Secondary endpoint (2°) investigated** | **Verum reaching 1°** | **Control reaching 1°** | **Verum reaching 2°** | **Control reaching 2°** | **Adverse Events** | **QoL** | **Serious adverts events** |
| Stone et al., 2010 (RAVE) | 197 | 375mg/m^2^ weekly for 4 weeks | Remission of disease (BVAS/WG of 0) with successful corticosteroid taper at 6 months. | Rates of disease flares (increase in BVAS/WG of 1 point or more), BVAS/WG of 0 during treatment with corticosteroid at a dose of less than 10mg/d, cumulative glucocorticoid doses, rates of AEs, and SF-36 scores. | 64% of patients reached disease remission (p=0.09). | 53% of patients reached disease remission. | No information. | No information. | 1035 AEs | QoL outcomes did not differ significantly. | 79 SAEs and ≥grade 3 AEs. 1 death. |

Abbreviations: ACR, American college of Rheumatology; AE, adverse event; ALT, alanine aminotransferase; AST, aspartate aminotransferase; AZA, azathioprine; BVAS-WG, Birmingham vasculitis activity score for Wegener's granulomatosis; CHAQ, childhood health assessment questionnaire; CR, complete remission; CRP, C-reactive protein; CSF, cerebrospinal fluid; CYC, cyclophosphamide; DLQI, dermatology quality of life index; EDSS, expanded disability disease scale; eGFR, epidermal growth factor receptor; ESRD, end-stage renal disease; FACIT-F, functional assessment of chronic illness therapy-Fatigue; HAQ-DI, Health Assessment Questionnaire Disability Index; IV, intravenous; IVIG, intravenous immunoglobulin; KS, Kaposi's sarcoma; MCD, minimal change disease; mTSS, Genant-modified sharp score; MTX, methotrexate; PLEX, plasma exchange; PR, partial remission; PsARC, Psoriatic arthritis response criteria; QoL, quality of life; RBV, ribavirin; SAE, serious adverse event; SR, sustained response; TADAI, total adjusted disease activity index; VAS, visual analogue scale.

| **Source** | **Patients treated** | **RTX** | **Primary endpoint (1°) investigated** | **Secondary endpoint (2°) investigated** | **Verum reaching 1°** | **Control reaching 1°** | **Verum reaching 2°** | **Control reaching 2°** | **Adverse Events** | **QoL** | **Serious adverts events** |
| --- | --- | --- | --- | --- | --- | --- | --- | --- | --- | --- | --- |
| Specks et al., 2013 (extension of RAVE) | 160 | 375mg/m^2^ weekly for 4 weeks, followed by placebo. | Remission of disease (BVAS/WG of 0) with successful corticosteroid taper at 6 months and maintaining remission through 12 and 18 months. | No information. | 39% of patients maintained complete remission for 18 months. | 33% of patients maintained complete remission for 18 months. | No information. | No information. | 98 patients (99%) with at least one AE. | No information. | 42 patients with at least one SAE. 2 deaths: sepsis with multi-organ failure and respiratory failure. |
| Miloslavsky et al., 2014 (open-label extension of RAVE) | 26 | Patients with a severe relapse between months 6 and 18 received open-label RTX in combination with IV corticosteroid. | Remission of disease (BVAS/WG of 0) and corticosteroid dose of 0 at any time following retreatment with RTX and glucocorticoids. | Complete response (BVAS/WG of 0 with corticosteroid of maximum 10mg/d), number of disease relapses, severe relapses. | No information. | No control. | No information. | No control. | 156 AEs: 85 in 14 patients previously treated with RTX and 71 in 9 patients previously treated with CYC/AZA. | No information. | No information. |
| de Menthon et al., 2011 | 17 | 375mg/m^2^ was given on days 1, 8, 15, and 22. Patients with a response, received further infusions at 4, 8, and 12 months. | Number of PR of clinical manifestations and BVAS decrease of >50% or CR of active vasculitis manifestation, BVAS=0 obtained after 12 months. | Treatment tolerance, side effects. | 62.5% of patients achieved either CR or PR. | 33% of patients achieved either CR or PR. | No information. | No information. | No allergic reactions and no severe infections. No significant difference in side effects was observed. | No information. | 1 death (sudden death at home after 4th infusion). No clear information about SAEs. |
| Guillevin et al., 2014 (MAINRITSAN) | 115 | 500mg on days 0 and 14 and at 6, 12, and 18 months. | Percentage of patients with major relapse at month 28 (reappearance or worsening of disease with a BVAS >0 and involvement of at least one major organ, a life-threatening manifestation, or both). | Rates of minor relapses (reappearance or worsening of disease with a BVAS >0, not corresponding to a major relapse but requiring mild treatment intensification), rates of adverse events, mortality | 5% of patients experienced a major relapse (p=0.002). | 29% of patients experienced a major relapse. | 11% of patients with a minor relapse (p=0.43). | 16% of patients with a minor relapse. | No information. | No information. | No information on SAEs. No deaths. |
| Charles et al., 2018 (MAINRITSAN2) | 162 (117 with GPA, 45 with MPA). | 500mg at randomization and every 3 months thereafter if ANCA status differed from previous control or CD19^+^ B cell count exceeded 0/mm3. | Number of relapses at month 28 (relapse = BVAS >0). | Number of major relapses (life-threatening or involving at least one major organ), number of minor relapses, association of ANCA evolution/ CD19^+^ B cell count with relapses, glucocorticoid duration, glucocorticoid cumulative dose. | 17.3% with a relapse (p=0.22). | 9.9% with a relapse. | Relapse-free survival rate: 83.8%. | Relapse-free survival rate: 86.4%. | 69 patients (85.2%) with at least one AE. | No information. | 26 patients (32.1%) with at least one SAE. 1 patient died (bronchospasm). |

| **Source** | **Patients treated** | **RTX** | **Primary endpoint (1°) investigated** | **Secondary endpoint (2°) investigated** | **Verum reaching 1°** | **Control reaching 1°** | **Verum reaching 2°** | **Control reaching 2°** | **Adverse Events** | **QoL** | **Serious adverts events** |
| --- | --- | --- | --- | --- | --- | --- | --- | --- | --- | --- | --- |
| Jones et al., 2010 (RITUXVAS) | 44 | 375mg/m^2^ weekly for 4 weeks with 2 IV CYC pulses of 15mg/kg with the first and the third RTX infusion. | Sustained remission (BVAS of 0 for at least 6 months) and rates of SAE (CTCAE grade ≥3) at 2 years. | Time to remission, change in BVAS between 0 and 3 months, change in GFR, change in corticosteroid dose, score on the SF-36 questionnaire, score on the Vasculitis Damage Index. | 76% of patients reached sustained remission (p=0.68). | 82% of patients reached sustained remission. | 91% of patients reached remission. | 91% of patients reached remission. | 25 patients (76%) experienced at least 1 AE. | The change in the score on the physical component of the SF-36 did not differ significantly. The control group showed a significant better outcome in the mental component of the SF-36 (p=0.04). | 16 patients (48%) with at least one SAE. 6 deaths: 3 due to infections, 1 due to cardiovascular disease, 2 due to complications of end-stage renal failure. |
| Jones et al., 2015 (extension of RITUXVAS) | 44 | 375mg/m^2^ weekly for 4 weeks with 2 IV CYC pulses of 15mg/kg with the first and the third RTX infusion. | Composite of death, ESRD, relapse at 24 months. | Time to death, ESRD and relapse. | 42% reached the primary endpoint (p=1.00). | 36% reached the primary endpoint. | Cumulative ESRD and relapse-free survival at 24 months: 58% (p=0.37). Cumulative survival rate at 24 months: 82% (p=0.56). | Cumulative ESRD and relapse-free survival at 24 months: 73%. Cumulative survival rate at 24 months: 73%. | 27 patients (82%) with at least one AE. | No information. | 20 patients (61%) with at least one SAE. 6 deaths. |
| Berden et al., 2012 (substudy of RITUXVAS) | 30 | 375mg/m^2^ weekly for 4 weeks with 2 IV CYC pulses of 15mg/kg with the first and the third RTX infusion. | eGFR during follow up (6, 12, and 24 months). | Time to clinical remission (BVAS score of 0 for at least 2 months and absence of clinical disease activity). | No information. | No information. | No information. | No information. | No information. | No information. | No information. |

| **Antiphospholipid syndrome** | | | | | | | | | | | | |
| --- | --- | --- | --- | --- | --- | --- | --- | --- | --- | --- | --- | --- |
| **Source** | **Patients treated** | **RTX** | **Primary endpoint (1°) investigated** | **Secondary endpoint (2°) investigated** | **Verum reaching 1°** | **Control reaching 1°** | **Verum reaching 2°** | **Control reaching 2°** | **Adverse Events** | **QoL** | **Serious adverts events** | |
| Erkan et al., 2013 (RITAPS) | 19 | 1000mg on days 1 and 15 | Safety and tolerability up to 12 months | Efficacy for non-criteria manifestations of antiphospholipid antibody for up to 6 months and effect on antiphospholipid antibody profile for up to 12 months | See adverse events | No control. | No information. | No control. | 49 AEs: 8 within the first 24 hours, 41 more 24h after the infusion. | No significant change in the SF-36 scores and PGA score at 24 weeks. | 12 SAEs: 5 neurologic, 3 infectious, 2 vascular, 1 cardiac and 1 allergic. No information on deaths. | |
| **Autoimmune haemolytic anaemia** | | | | | | | | | | | |  |
| **Source** | **Patients treated** | **RTX** | **Primary endpoint (1°) investigated** | **Secondary endpoint (2°) investigated** | **Verum reaching 1°** | **Control reaching 1°** | **Verum reaching 2°** | **Control reaching 2°** | **Adverse Events** | **QoL** | **Serious adverts events** |  |
| Birgens et al., 2013 | 64 | 375mg/m^2^ weekly for 4 weeks in combination with corticosteroid. | Treatment response after 3, 6, and 12 months: CR (normalization in haemoglobin concentration without any ongoing immunosuppressive treatment and without any biochemical signs of haemolytic activity), PR (similar to CR but requiring low-dose corticosteroid (<10mg/d), or appearing as compensated haemolytic anaemia entailing a stable, acceptable haemoglobin level without any need of treatment except low-dose corticosteroid). | Relapse-free survival, red blood cell transfusion requirement, need for splenectomy, and safety profiles. | 3 months: CR or PR response in about 50% of patients (p=0.7). 6 months: CR in 63% (p=0.07). 12 months: CR in 75% of patients (p<0.003). | 3 months: CR or PR response in 50% of patients. 6 months: CR in 39%. 12 months: CR in 36% of patients. | No information. | No information. | No significant difference in the occurrence of AEs. | No information. | 9 patients with an SAE (5 non-fatal and 4 with fatal outcome). |  |
| **Source** | **Patients treated** | **RTX** | **Primary endpoint (1°) investigated** | **Secondary endpoint (2°) investigated** | **Verum reaching 1°** | **Control reaching 1°** | **Verum reaching 2°** | **Control reaching 2°** | **Adverse Events** | **QoL** | **Serious adverts events** |  |
| Michel et al., 2016 (RAIHA) | 32 | 1000mg on days 1 and 15 | Overall response rate (CR + PR) at 1 year: CR = hemoglobin level ≥11g/dl or 12 g/dl without features of ongoing haemolysis, without any ongoing treatment for AIHA on 2 different occasions 4 weeks apart in the absence of any recent transfusion. PR = hemoglobin level ≥10g/dl with at least a 2g increase from baseline without any other treatment than corticosteroid ≤10mg/d or recent transfusion. | PR, cumulative dose of steroids, number of transfusions and hospitalizations at 1 year, incidence and severity of AEs, CR/PR at 2 years. | Overall response rate: 75% (11 CR, 1 PR) (p=0.032). | Overall response rate: 31% (CR 5). | No information. | No information. | No information. | No information. | 4 nonfatal SAEs in 3 patients. No deaths. |  |
| **Autoimmune hepatitis** | | | | | | | | | | | |  |
| **Source** | **Patients treated** | **RTX** | **Primary endpoint (1°) investigated** | **Secondary endpoint (2°) investigated** | **Verum reaching 1°** | **Control reaching 1°** | **Verum reaching 2°** | **Control reaching 2°** | **Adverse Events** | **QoL** | **Serious adverts events** |  |
| Burak et al., 2013 | 6 | 1000mg on days 1 and 15 | Safety (lack of infusion reactions or SAE) over 72 weeks | Efficacy at week 24: change in ALT, AST, bilirubin, gammaglobulin and IgG levels as well as FSS | No information. | No control. | No information. | No control. | Mild unilateral conjunctivitis and dental abscess in one patient. One patient developed jaundice and elevated ALT levels which was considered as a disease flare. | No significant change in the nine-point fatigue severity scale. | No SAEs. |  |
| **Behçet’s disease** | | | | | | | | | | | |  |
| **Source** | **Patients treated** | **RTX** | **Primary endpoint (1°) investigated** | **Secondary endpoint (2°) investigated** | **Verum reaching 1°** | **Control reaching 1°** | **Verum reaching 2°** | **Control reaching 2°** | **Adverse Events** | **QoL** | **Serious adverts events** |  |
| Davatchi et al., 2010 | 20 | 1000mg on days 1 and 15 in combination with MTX and corticosteroid | Overall state of patient's eyes calculated by TADAI | Visual acuity, posterior uveitis, and retinal vasculitis | No information. | No information. | No information. | No information. | 2 patients with conjunctivitis, 1 patient with pneumonia, and 1 patient with herpes zoster. Further side effects comprised infusion related reactions (1 mild urticaria, 1 flushing and 1 severe allergic reaction). | No information. | No information. |  |

| **Bullous pemphigoid** | | | | | | | | | | | |
| --- | --- | --- | --- | --- | --- | --- | --- | --- | --- | --- | --- |
| **Source** | **Patients treated** | **RTX** | **Primary endpoint (1°) investigated** | **Secondary endpoint (2°) investigated** | **Verum reaching 1°** | **Control reaching 1°** | **Verum reaching 2°** | **Control reaching 2°** | **Adverse Events** | **QoL** | **Serious adverts events** |
| Hall et al., 2013 | 7 | 1000mg on days 0 and 14. | No primary endpoint defined. | No secondary endpoints defined. | No information. | No control. | No information. | No control. | No information. | No information. | No SAE. |

| **Castleman’s disease** | | | | | | | | | | | |
| --- | --- | --- | --- | --- | --- | --- | --- | --- | --- | --- | --- |
| **Source** | **Patients treated** | **RTX** | **Primary endpoint (1°) investigated** | **Secondary endpoint (2°) investigated** | **Verum reaching 1°** | **Control reaching 1°** | **Verum reaching 2°** | **Control reaching 2°** | **Adverse Events** | **QoL** | **Serious adverts events** |
| Marcelin et al., 2003 | 5 | 375mg/m^2^ weekly for 4 weeks. | No primary endpoint defined. | No secondary endpoints defined. | No information. | No control. | No information. | No control. | 2 patients with aggravation of KS | No information. | No SAEs. 2 deaths due to severe hematologic autoimmune disorders associated with MCD. |
| Ide et al., 2006 | 3 | 375mg/m^2^ weekly for 4 or 8 weeks, followed by oral corticosteroid and/or alkylating agents for maintenance therapy | No primary endpoint defined | No secondary endpoints defined | No information. | No control. | No information. | No control. | No adverse events | No information. | No SAEs. |
| Bower et al., 2007 | 21 | 375mg/m^2^ weekly for 4 weeks | No primary endpoint defined | No secondary endpoints defined | No information. | No control. | No information. | No control. | 4/11 patients had aggravation of KS. No further information. | No information. | 1 death due to progressive disease. |

| **Source** | **Patients treated** | **RTX** | **Primary endpoint (1°) investigated** | **Secondary endpoint (2°) investigated** | **Verum reaching 1°** | **Control reaching 1°** | **Verum reaching 2°** | **Control reaching 2°** | **Adverse Events** | **QoL** | **Serious adverts events** |
| --- | --- | --- | --- | --- | --- | --- | --- | --- | --- | --- | --- |
| Gerard et al., 2007 | 24 | 375mg/m^2^ weekly for 4 weeks | Sustained remission rate at day 60 (absence of MCD relapse) off chemotherapy. Mild symptoms were accepted if the symptoms did not require chemotherapy and if they resolved before day 29). | Persistence of SR at day 365, disease-free survival, event-free survival, overall survival, occurrence of lymphoma, occurrence or exacerbation of KS, safety, evolution of human herpesvirus -8 viral load and B cell count. | 92.0% | No control. | Overall survival rate after 1 year: 92%. Event-free survival after 1 year: 71%. Disease-free survival after 1 year: 77%. Sustained remission after 1 year: 71%. | No control. | 9 patients experienced an RTX related AE. Most common: chills, fever, headache, pruritus, rash, dizziness, hypotension, and infections. 8 patients experienced exacerbation of KS lesion. | No information. | 2 deaths: One due to progressive disease and one due to acute respiratory failure of undetermined origin. |
| Powles et al., 2007 | 3 | Unknown dosing regimen | No primary endpoint defined | No secondary endpoints defined | No information. | No control. | No information. | No control. | No information. | No information. | No information. |
| Bestawros et al., 2008 | 4 | 375mg/m^2^ weekly for 4 weeks | No primary endpoint defined | No secondary endpoints defined | No information. | No control. | No information. | No control. | No information. | No information. | 1 death due to progressive disease. |
| Peker et al., 2012 | 4 | Unknown dosing regimen | No primary endpoint defined | No secondary endpoints defined | No information. | No control. | No information. | No control. | No information. | No information. | No information. |
| Uldrick, T.S., 2014 | 17 | RTX 375mg/m2 every 3 weeks up to 2 cycles beyond resolution of symptoms and marked improvement in biochemical abnormalities. Followed by a consolidation therapy with either interferon-alpha or AZT in combination with valganciclovir. | No primary endpoint defined | No secondary endpoints defined | 15/17 (88%) patients achieved a complete response | No control | Patients received a median of 4 cycles (range 3-9). 15 received consolidation therapy. Event-free survival: 81.6% at 12 months and 69% at 24 months. Overall survival: 93.8% at 1 year, 87.5% at 2 years, 81% at 3 years. Significant improvement of KSHV viral load, IL-6, IL-10, hemoglobin, albumin, and C-reactive protein (CRP). Increase in CD4 cells (p=0.07), marked decrease in CD19 B cells. | No control | Overall R-Dox was well tolerated. Most common AE: infusion reaction (70% of patients). | No information | No information. |
| **Cryoglobulinemia** | | | | | | | | | | | |
| **Source** | **Patients treated** | **RTX** | **Primary endpoint (1°) investigated** | **Secondary endpoint (2°) investigated** | **Verum reaching 1°** | **Control reaching 1°** | **Verum reaching 2°** | **Control reaching 2°** | **Adverse Events** | **QoL** | **Serious adverts events** |
| Dammacco et al., 2010 | 37 | 375mg/m^2^ weekly for 4 weeks followed by 2 5-monthly infusions accompanied by Peg-IFN-alpha2a weekly in combination with RBV. | Proportion of patients with a complete response (disappearance or remarkable improvement of clinical features, disappearance of cryoglobulins, detectability of serum Hepatitis C virus RNA, and disappearance of B cell clonalities from the blood) after 1 year. | No information. | 54.5% of patients (p<0.05). | 33.3% of patients. | Response maintenance rate after 36 months: 83.3% (p<0.01). | Response maintenance rate after 36 months: 40%. | 3 patients with a mild AE after first infusion. 2 patients with fever after third and fourth infusion. | No information. | No deaths. |
| De Vita et al., 2012 | 57 | 1000mg on days 0 and 14. | Proportion of patients continuing taking their initial therapy after 12 months. | Proportion of patients continuing taking their initial therapy after 3 months, 6 months, and 24 months, BVAS, CV manifestations, duration of treatment response, efficacy of retreatment, and side effects. | 64.3% (p<0.0001). | 3.5% of patients. | Survival of treatment after 3 months: 92.9% (p<0.0001), 6 months: 71.4% (p<0.0001) 24 months: 60.7% (p<0.0001). | Survival of treatment after 3 months: 13.8%. 6 months: 3.5%. 24 months: 3.5%. | No information. | No information. | 11 SAE (3 infections, 5 cardiovascular events, 1 acute liver failure, 1 gastrointestinal bleeding, 1 hemorrhagic alveolitis), 5 deaths: 1 acute liver failure, 1 myocardial infarction, 2 heart failure and 1 hemorrhagic alveolitis. |

| **Source** | **Patients treated** | **RTX** | **Primary endpoint (1°) investigated** | **Secondary endpoint (2°) investigated** | **Verum reaching 1°** | **Control reaching 1°** | **Verum reaching 2°** | **Control reaching 2°** | **Adverse Events** | **QoL** | **Serious adverts events** |
| --- | --- | --- | --- | --- | --- | --- | --- | --- | --- | --- | --- |
| Quartuccio et al., 2015 (Follow-up of De Vita et al., 2012) | 30 | 1000mg given two weeks apart in case of clinical relapse. | No information. | No information. | 57.1% in remission, 23.8% with partial response, 19% with active disease | No control. | No information. | No control. | 9/30 patients had at least one AE. | No information. | 6 deaths: 2 intestinal vasculitis, 2 heart failure, 1 liver failure in hepatocellular carcinoma and 1 unknown cause. |
| Sneller et al., 2012 | 24 | 375mg/m^2^ weekly for 4 weeks. | Number of patients in remission (BVAS of 0) at 6 months. | Duration of remission, SAEs. | 83.3% of patients (p<0.001). | 8.3% of patients. | No information. | No information. | No information. | No information. | 1 SAE: grade 4 fever. No deaths. |
| **Goodpasture’s syndrome** | | | | | | | | | | | |
| **Source** | **Patients treated** | **RTX** | **Primary endpoint (1°) investigated** | **Secondary endpoint (2°) investigated** | **Verum reaching 1°** | **Control reaching 1°** | **Verum reaching 2°** | **Control reaching 2°** | **Adverse Events** | **QoL** | **Serious adverts events** |
| Shah et al., 2012 | 3 | 375mg/m^2^ weekly for 4 weeks (2 weeks respectively in Case 3) | No primary endpoint defined | No secondary endpoints defined | No information. | No control. | No information. | No control. | No complications occurred. | No information. | No deaths. |

| **Grave's disease** | | | | | | | | | | | |
| --- | --- | --- | --- | --- | --- | --- | --- | --- | --- | --- | --- |
| **Source** | **Patients treated** | **RTX** | **Primary endpoint (1°) investigated** | **Secondary endpoint (2°) investigated** | **Verum reaching 1°** | **Control reaching 1°** | **Verum reaching 2°** | **Control reaching 2°** | **Adverse Events** | **QoL** | **Serious adverts events** |
| Salvi et al., 2015 | 31 | 1000mg given twice two weeks apart. After 12 patients the treatment protocol was changed to RTX 500mg given once. | Reduction in clinical activity score of 2 points or to less than 3 at 24 weeks. | Changes of proptosis, lid fissure, diplopia, and eye muscle motility, as well as QoL. | Disease inactivation (CAS<3): 100% (p=0.43) | Disease inactivation (CAS<3): 68.7% | No information. | No information. | 13/15 patients (86.6%)experienced an AE | Significant improvement on the appearance scale and on the visual functioning scale at weeks 52 and 76 for the RTX group. ivMP patients only showed a significant improvement at 12 weeks in the appearance scale. | No information. |
| Stan et al., 2015 | 25 | 1000mg given twice two weeks apart. | Reduction in clinical activity score at 24 weeks. | Success rates, failure rates, proportions showing clinically significant improvement in proptosis, lid fissure width, diplopia score, lagophthalmos, and disease severity, as well as QoL and orbital fat/muscle volume. | No information. | No information. | 69% of patients failed therapy (p=0.75) | 75% of patients failed therapy. | 8/13 patients (61.5%) with at least one AE. | No significant differences in the QOL scores (SF-12 for physical and mental health) or their changes between the RTX and placebo group. | No information. |

| **IgA nephropathy** | | | | | | | | | | | |
| --- | --- | --- | --- | --- | --- | --- | --- | --- | --- | --- | --- |
| **Source** | **Patients treated** | **RTX** | **Primary endpoint (1°) investigated** | **Secondary endpoint (2°) investigated** | **Verum reaching 1°** | **Control reaching 1°** | **Verum reaching 2°** | **Control reaching 2°** | **Adverse Events** | **QoL** | **Serious adverts events** |
| Lafayette et al., 2016 | 34 | 1000mg on weeks 0 and 2 with a repeated course at 6 months. | Change in proteinuria and eGFR from baseline to 12 months. | Safety | No information. | No information. | No information. | No information. | AEs were more common in the RTX group. | No information. | No SAEs. No information on deaths. |
| **IgG4 related diseases** | | | | | | | | | | | |
| **Source** | **Patients treated** | **RTX** | **Primary endpoint (1°) investigated** | **Secondary endpoint (2°) investigated** | **Verum reaching 1°** | **Control reaching 1°** | **Verum reaching 2°** | **Control reaching 2°** | **Adverse Events** | **QoL** | **Serious adverts events** |
| Khosroshahi et al., 2010 | 4 | 1000mg twice given two weeks apart (except patient 3: interval was 1 month) | No primary endpoint defined | No secondary endpoints defined | No information. | No control. | No information. | No control. | No information. | No information. | No information. |
| Khosroshahi et al., 2012 | 10 | 1000mg twice given two weeks apart. | No primary endpoint defined | No secondary endpoints defined | 90% of patients experienced substantial clinical improvement within 1 month of therapy. | No control. | 90% of patients achieved disease response defined as improvement in IgG4-related disease Activity Index >2points. | No control. | 2 major AEs: one patient with flare of asthma requiring hospitalization and one patient with reactivation of HBV. No further information | No information. | No information. |
| Carruthers et al., 2015 | 30 | 1000mg twice given 2 weeks apart | Decline of the IgG4-RD Responder Index ≥2 points by month 6, no disease flares before month 6, and no glucocorticoid use between months 2 and 6. | No secondary endpoints defined | 77% of patients achieved the primary endpoint | No control. | No information. | No control. | No information. | Significant decrease in PGA (p<0.00001). 23/30 patients achieved a PGA score of 0. | 6 hospitalizations: Klebsiella urinary tract infection, Legionella pneumophila pneumonia, cold agglutinin-mediated haemolytic anaemia (no relation to RTX), amaurosis fugax, unstable angina (no relation to RTX), transitional cell cancer of the bladder (no relation to RTX), and orbital pseudotumor. |
| Yamamoto et al., 2015 | 3 | 500mg at the onset of relapse | No primary endpoint defined | No secondary endpoints defined | No information. | No control. | No information. | No control. | One patient experienced an AE: herpes zoster | No information. | No SAE, no death |
| Quattrocchio et al., 2018 | 5 | 375mg/m^2^ weekly for 4 weeks. Two more doses were given 1 and 2 months after the last infusion | No primary endpoint defined | No secondary endpoints defined | No information. | No control. | No information. | No control. | 2 patients developed steroid-induced diabetes mellitus and another patient bronchopneumonia. | No information. | No information. |

| **Immune thrombocytopenia** | | | | | | | | | | | |
| --- | --- | --- | --- | --- | --- | --- | --- | --- | --- | --- | --- |
| **Source** | **Patients treated** | **RTX** | **Primary endpoint (1°) investigated** | **Secondary endpoint (2°) investigated** | **Verum reaching 1°** | **Control reaching 1°** | **Verum reaching 2°** | **Control reaching 2°** | **Adverse Events** | **QoL** | **Serious adverts events** |
| Hasan et al., 2009 (only considering Part 2) | 16 | 750mg/m^2^ weekly for 4 weeks (double dose RTX group) | Higher response rates (more CR's and/or PR's) or longer response duration. CR = platelet count ≥150'000 for ≥3 months, PR = platelet count ≥50'000 for ≥3 months. | Response to treatment, time to response, and toxicity | No information. | No information. | No information. | No information. | Well tolerated. | No information | No information. |
| Zaja et al., 2010 | 101 | 375mg/m2 weekly for 4 weeks plus dexamethasone 40mg orally on days 1-4. | Sustained response (SR) rate (=platelet count ≥50 x10^9/l at month 6) | Hematologic response (platelet count ≥100x10^9/l = SR100 and ≥150x10^9/l = SR150), safety profile, early response (platelet count ≥50x10^9/l at day28), efficacy of salvage therapy with dexa plus RTX, predictors of SR, and cellular and humoral immune response | 63% achieved SR (p=0.004) | 36% achieved SR | No information | No information | 37 patients (76%) experienced at least one AE. | No information | 3 patients (6%) with at least one SAE: 1 patient with hemorrhagic disorder (platelet decrease and/or bleeding), 1 patient with supraventricular tachycardia and 1 patient with interstitial pneumonia. The hemorrhagic disorder was considered as not related to study drug. No deaths. |
| Li et al., 2011 | 62 | 100mg weekly for 4 weeks with dexamethasone 40mg/d orally for 4 days followed by prednisone orally tapered consecutively. | No primary endpoint defined | No secondary endpoints defined | Overall response rate at day 28: 80.6% (p=0.938), Complete response rate: 67.7%, Partial response rate: 12.9% | Overall response rate at day 28: 74.2%, Complete response rate: 54.8%, Partial response rate: 19.4%. | Sustained response (platelet count ≥50x10^9/l at month 6 after initial treatment): 77.4% | Sustained response (platelet count ≥50x10^9/l at month 6 after initial treatment): 38.7% | 3/31 (9.6%) of the patients with at least one AE. | No information | No information about SAEs and deaths. |
| Arnold et al., 2012 | 58 | 375mg/m2 weekly for 4 weeks | Treatment failure (= any platelet count <50x10^9/l or significant bleeding defined as grade 2 severity or rescue treatment administration) | QoL (SF-36), proportion of patient with a complete response (platelet count ≥100x10^9/l) and overall response (platelet count ≥30x10^9/l with doubling from baseline). | 65.6% of the patients met the primary endpoint | 80.8% of the patients met the primary endpoint | Complete response after 6 months: 53.1%. Overall response: 62.5%. | Complete response after 6 months: 46.2%. Overall response: 73.1%. | 76 AEs | No treatment effect for change in QoL scores for physical or mental domains (SF-36) | 2 SAEs: serum sickness and accidental fall. |
| Gudbrandsdottir et al., 2013 | 133 | 375mg/m2 weekly for 4 weeks, DXM 40mg/d orally for 4 consecutive days. | Sustained partial or complete response at 6 months (CR= platelet count ≥100x10^9/l, PR= platelet count ≥50x10^9/l) | Time to relapse, time to rescue treatment, and rates of splenectomy | 58% of the patients achieved SR (p=0.02) | 37% of the patients achieved SR | No information | No information | AEs were mostly mild and balanced between the 2 groups. | No information | 16 SAE including 1 death. |
| Dai et al., 2015 | 50 | 375mg/m2 weekly for 4 weeks | No information | No information | Overall response rate: 69.2% (p<0.01). Complete response (=platelet count >150x10^9/l, no bleeding symptoms): 42.3%. Partial response (=platelet count elevated, improved bleeding symptoms): 26.9% | Overall response rate: 37.5%. Complete response (=platelet count >150x10^9/l, no bleeding symptoms): 12.5%. Partial response (=platelet count elevated, improved bleeding symptoms): 25% | No information | No information | 3 children with an AE: one mild rash, 2 cases of abnormal liver and kidney function. | No information | No SAEs. No information on deaths. |
| Ghanima et al., 2015 (RITP trial) | 109 | 375mg/m2 weekly for 4 weeks | Rate of treatment failure (=splenectomy or meeting the criteria for splenectomy after week 12 [=platelet count lower than 20x10^9/l or need for prednisone increments at doses higher than 7.5mg/d to maintain platelet count of less than or equal to 20x10^9/l]) within 78 weeks. | Response rates (Overall response [OR] = platelet count ≥30x10^9/l, complete response [CR] = platelet count ≥100x10^9/l), relapse rates, duration of response, bleeding, safety, and corticosteroid consumption. | 58% of the patients had treatment failure (p=0.65) | 68% of the patients had treatment failure | OR after 78 weeks: 81% (p=0.15). CR after 78 weeks: 58% (p=0.12) | OR after 78 weeks: 73%. CR after 78 weeks: 50% | No information | No information | No information about SAEs. No deaths. |
| **Inflammatory myositis** | | | | | | | | | | | |
| **Source** | **Patients treated** | **RTX** | **Primary endpoint (1°) investigated** | **Secondary endpoint (2°) investigated** | **Verum reaching 1°** | **Control reaching 1°** | **Verum reaching 2°** | **Control reaching 2°** | **Adverse Events** | **QoL** | **Serious adverts events** |
| Oddis et al., 2013 (RIM trial) | 200 | RTX early arm: 575mg/m^2^ (children with body surface area ≤1.5m2) or 750mg/m^2^ (children with BSA>1.5m2) on weeks 0 and 1 with placebo on weeks 8 and 9. | Time to achieve definition of improvement (≥20% improvement in 3 of any 6 core set measures, with no more than 2 worsening by ≥ 25%). | Time to 20% improvement in the MMT-8, proportion of patients achieving definition of improvement at week 8. | No information. | No information. | Proportion of patients achieving DOI by week 8: 15%. | Proportion achieving DOI by week 8: 20.6%. | No difference in number of AEs at week 8 with the exception of infusion reactions which occurred significantly more often in the RTX group (p<0.01). | No information. | 64 patients experienced 67 SAEs (26 SAEs were considered as drug related SAE). 1 death: lung mass suspicious for malignancy followed by a stroke. |
| **Juvenile idiopathic arthritis** | | | | | | | | | | | |
| **Source** | **Patients treated** | **RTX** | **Primary endpoint (1°) investigated** | **Secondary endpoint (2°) investigated** | **Verum reaching 1°** | **Control reaching 1°** | **Verum reaching 2°** | **Control reaching 2°** | **Adverse Events** | **QoL** | **Serious adverts events** |
| Narvaez et al., 2009 | 3 | 1000mg on days 1 and 15 (one patient received 500mg during the first cycle due to weight of 40kg) | No information. | No information. | No information. | No control. | No information. | No control. | Patient 1 experienced a hypersensitivity reaction during the second infusion of the second treatment cycle. | No information. | No SAE, no death |
| Alexeeva et al., 2011 | 55 | 375mg/m^2^ weekly for 4 weeks. Further treatment courses if systemic manifestations persisted at 24 weeks. | ACR Pedi 30 response at week 24. | ACR Pedi 50 and 70 responses and clinical remission at weeks 24, 48, 72, and 96. | 98% of patients achieved ACR Pedi30 at 24 weeks. | No control. | At 48 weeks: 75% of patients achieved ACR50 response and 70% ACR70 response. Clinical and laboratory remission was reported in 52%. At 52 weeks: 75% of patients achieved ACR 50 and 70 responses and clinical and laboratory remission. At 96 weeks: 93% of patients achieved ACR70 response. | No control. | 101 AEs were reported with the most common AE being transfusion reactions (36 events). | Significant improvement in the CHAQ score after 12 weeks (p<0.001). | No information. |

| **Membraneous nephropathy** | | | | | | | | | | | |
| --- | --- | --- | --- | --- | --- | --- | --- | --- | --- | --- | --- |
| **Source** | **Patients treated** | **RTX** | **Primary endpoint (1°) investigated** | **Secondary endpoint (2°) investigated** | **Verum reaching 1°** | **Control reaching 1°** | **Verum reaching 2°** | **Control reaching 2°** | **Adverse Events** | **QoL** | **Serious adverts events** |
| Dahan et al., 2017 | 75 | 375mg/m2 on days 1 and 8 | Percentage of patients with complete or partial remission of nephrotic syndrome at 6 months (complete remission= urinary protein excretion <500mg/d or <500mg/g creatinine. Partial remission = urinary protein excretion <3.5gr/d or <3500mg/g creatinine and ≥500mg/g creatinine with ≥50% reduction compared with baseline) | Rate of proteinuria, serum albumin, serum creatinine, anti-phospholipase A2 receptor antibody levels, SAEs | 35.1% (p=0.21) | 0.21 | Significant reduction in PLA2R-Ab titers (p<0.001). Significant more patients with a remission during follow-up (64.9%, p<0.01) | Significant less patients with a remission during follow-up (34.2%) | No information. | No information. | 6 patients with at least one SAE |

| **Multiple sclerosis** | | | | | | | | | | | |
| --- | --- | --- | --- | --- | --- | --- | --- | --- | --- | --- | --- |
| **Source** | **Patients treated** | **RTX** | **Primary endpoint (1°) investigated** | **Secondary endpoint (2°) investigated** | **Verum reaching 1°** | **Control reaching 1°** | **Verum reaching 2°** | **Control reaching 2°** | **Adverse Events** | **QoL** | **Serious adverts events** |
| Hauser et al., 2008 | 104 | 1000mg on days 1 and 15 | Total count of gadolinium-enhancing lesions detected on T1-weighted MRI scans of the brain at weeks 12, 16, 20, and 24 | Proportion of patients with relapses, annualized rate of relapse, and total number of new gadolinium-enhancing lesions on T1-weighted MRI scans at weeks 12, 16, 20, and 24, and the change from the baseline lesion volume on T2-weighted MRI scans. | Relative reduction of gadolinium-enhancing lesions of 91%. | No information. | Number of patients with a relapse at week 24: 14.5% (p=0.02) and week 48: 20.3% (p=0.04) | Patients with a relapse at week 24: 34.3% and at week 48: 40.0% | 68 patients (98.6%) with at least one AE. | No information. | 13% experienced a SAE. 1 death: homicide |
| Hawker et al., 2009 | 439 | 1000mg on days 1 and 15 with repeated courses every 24 weeks | Time to confirmed disease progression (=EDSS increase of ≥1.0 point from baseline if EDSS was between 2.0 and 5.5, or and EDSS increase of ≥0.5 if EDSS was >5.5 sustained for at least 12 weeks). | Change from baseline to week 96 in T2 lesion volume and total brain volume on MRI scans. | confirmed disease progression rate: 30.2% | confirmed disease progression rate: 38.5% | No information. | No information. | 289 patients (99%) experienced at least one AE. | No information. | 48 patients (16.4%) with at least one SAE. 1 death due to aspiration and consecutive pneumonia owing to brainstem lesions. |
| Komori et al., 2016 (RIVITALISE trial) | 27 | RTX 25mg intrathecal twice given at months 0, 1.5 and 12. RTX 200mg IV was given after intrathecal application at months 0 and 0.5 and 2 weeks later. | No primary endpoint defined | No secondary endpoint defined | No information | No information | Significant increase in BAFF in serum and cerebrospinal fluid (CSF). Significant decrease of CXCL13 in serum, but not in CSF. | No information | No information | No information | No information |

| **Myasthenia gravis** | | | | | | | | | | | |
| --- | --- | --- | --- | --- | --- | --- | --- | --- | --- | --- | --- |
| **Source** | **Patients treated** | **RTX** | **Primary endpoint (1°) investigated** | **Secondary endpoint (2°) investigated** | **Verum reaching 1°** | **Control reaching 1°** | **Verum reaching 2°** | **Control reaching 2°** | **Adverse Events** | **QoL** | **Serious adverts events** |
| Illa et al., 2008 | 6 | 375mg/m^2^ weekly for 4 weeks and monthly thereafter | No primary endpoint defined | No secondary endpoint defined | All patients showed a significant reduction in antibody titers as well as IgG concentrations (p=0.006 and p=0.04). | No control. | No information. | No control. | No AEs occurred | No information. | No SAEs. No information of deaths. |
| Lebrun et al., 2009 | 6 | 375mg/m^2^ weekly for 4 weeks and monthly thereafter | No primary endpoint defined | No secondary endpoint defined | 5 patients were able to stop corticosteroid after 1 year of treatment and cholinesteratic inhibitors were tapered off to 60-180mg/d. | No control. | No information. | No control. | No AEs occurred | No information. | No information. |
| Nelson et al., 2009 | 3 | 375mg/m^2^ weekly for 4 weeks | No primary endpoint defined | No secondary endpoint defined | No information. | No control. | No information. | No control. | No AEs occurred | No information. | No information. |
| Stieglbauer et al., 2009 | 3 | 375mg/m^2^ weekly for 2 weeks. Additional single infusions were given if B cells reappeared. | No primary endpoint defined | No secondary endpoint defined | No information. | No control. | No information. | No control. | No information. | No information. | No information. |
| Lindberg et al., 2010 | 5 | 375mg/m^2^ weekly for 4 weeks. Some patients received retreatment with 2 infusions RTX 1000mg given 2 weeks apart if clinical symptoms worsened. | No primary endpoint defined | No secondary endpoint defined | No information. | No control. | No information. | No control. | No information. | No information. | One patient with a RTX-related SAE: agranulocytosis and pneumonia. Patient 3 died due to heart failure. |

| **Source** | **Patients treated** | **RTX** | **Primary endpoint (1°) investigated** | **Secondary endpoint (2°) investigated** | **Verum reaching 1°** | **Control reaching 1°** | **Verum reaching 2°** | **Control reaching 2°** | **Adverse Events** | **QoL** | **Serious adverts events** |
| --- | --- | --- | --- | --- | --- | --- | --- | --- | --- | --- | --- |
| Diaz et al., 2012 | 17 (6 MuSK patients, 11 AChR patients) | 375mg/m^2^ weekly for 4 weeks and monthly applications thereafter. Infusions were repeated if symptoms reappeared. | No primary endpoint defined. | No secondary endpoint defined. | Peripheral B cells were completely depleted in all patients. | No control. | No information. | No control. | 2 patients experienced an AE: facial flushing and generalized skin rush. Both resolved after parenteral hydrocortisone and diphenhydramine. | No information. | No information. |
| Sun et al., 2014 | 22 | 375mg weekly for 4 weeks. Additional infusions were administered when CD19 B cells exceeded 1%. | No primary endpoint defined. | No secondary endpoint defined. | Compared with healthy subjects, MG patients had significantly less CD19^+^ B cells, that produce IL-10 (p<0.05). Furthermore patients with severe disease tend to have lower levels of IL-10 B cells (p<0.05). Non-responders to RTX treatment showed a delayed IL-10 B cell repopulation (p<0.05). Patients with anti-MuSK antibodies tended to have a faster IL-10 B cell recovery than patients with anti-AChR antibodies. | No control. | No information. | No control. | No information. | No information. | No information. |
| **Source** | **Patients treated** | **RTX** | **Primary endpoint (1°) investigated** | **Secondary endpoint (2°) investigated** | **Verum reaching 1°** | **Control reaching 1°** | **Verum reaching 2°** | **Control reaching 2°** | **Adverse Events** | **QoL** | **Serious adverts events** |
| Anderson et al., 2016 | 14 | 375mg/m^2^ weekly for 4 or 750mg/m^2^ every two weeks for 1 month. | Change in manual muscle testing score. | Change in steroid dose and change in frequency of intravenous immunoglobulin (IVIG) infusions or plasma exchange (PLEX). | No information. | No control. | No information. | No control. | 3 patients experienced post-infusion headaches. | No information. | No SAEs, no deaths. |
| Beecher et al., 2018 (extension trial of Anderson et al., 2016) | 22 (9 additional patients were enrolled, all receiving regimen 2) | 375mg/m^2^ weekly for 4 weeks followed by monthly applications during the next 2 months (induction regimen 1) or 750mg/m^2^ every two weeks for 1 month (induction regimen 2) | Change in manual muscle testing score. | Change in steroid dose and change in frequency of IVIG infusions or PLEX. | No information. | No control. | No information. | No control. | 3 patients experienced mild post-infusion headache | No information. | No SAEs, one death. |
| Lebrun et al., 2016 | 24 | 375mg/m^2^ weekly for 4 weeks followed by infusions every 3 months (group 1). After 2013 patients received only additional infusions if CD27 expressing B cells were above 0.05% in PBMC (group 2). | No primary endpoint defined | No secondary endpoint defined | No information. | No control. | No information. | No control. | No information. | No information. | No SAEs. |
| Jing et al., 2017 | 8 | 100mg on the first day, 500mg on the second day. Cycles could be repeated every 6 months | No primary endpoint defined. | No secondary endpoint defined. | No information. | No control. | No information. | No control. | No clear information. | See primary endpoint. | No SAEs. |

| **Nephrotic syndrome** | | | | | | | | | | | |
| --- | --- | --- | --- | --- | --- | --- | --- | --- | --- | --- | --- |
| **Source** | **Patients treated** | **RTX** | **Primary endpoint (1°) investigated** | **Secondary endpoint (2°) investigated** | **Verum reaching 1°** | **Control reaching 1°** | **Verum reaching 2°** | **Control reaching 2°** | **Adverse Events** | **QoL** | **Serious adverts events** |
| Ravani et al., 2011 | 54 | 375mg/m^2^ once (absence of signs of toxicity secondary to steroids and/or calcineurin inhibitors) or twice 2 weeks apart (presence of toxicity). Beginning at 30 days, corticosteroid was tapered off if proteinuria was <1g/d. After 2 weeks calcineurin inhibitors were also decreased by 50% and withdrawn after 2 additional weeks. | Percentage change in daily proteinuria at 3 months. | Risk of relapse at 3 months (relapse= proteinuria >40mg/h or proteinuria of 20-40mg/h and hypoalbuinemia or dyslipidemia). | 70% reduction of mean proteinuria was observed in comparison to control group (p<0.003). | see verum 1. | Risk of disease relapse at 3 months: 18.5% (p=0.029). Probability of being corticosteroid-free at 3 months: 77.8% (p<0.001). Probability of being calcineurin- inhibitor-free at 3 months: 62.9% (p<0.001).56% of the children discontinued calcineurininhibitors (p<0.001). | Risk of disease relapse at 3 months: 48.1%. Probability of being corticosteroid-free at 3 months: 7.4%. Probability of being calcineurin-inhibitor-free at 3 months: 3.7%. 4% of the children discontinued calcineurin-inhibitors. | 1 patient with bronchospasm and hypotension. 2 patients with bronchospasm. 2 patients with fever and migrating skin rash and acute arthritis at the hip joint. | No information. | No information. |
| Ravani et al., 2013 (long-term follow-up of Ravani et al., 2011) | 46 | 375mg/m^2^ once (absence of signs of toxicity secondary to steroids and/or calcineurin inhibitors) or twice 2 weeks apart (presence of toxicity). Beginning at 30 days, corticosteroid was tapered off if proteinuria was <1g/d. After 2 weeks calcineurin inhibitors were also decreased by 50% and withdrawn after 2 additional weeks. | Probability of maintaining remission (absence of proteinuria) for at least 6 months after successful tapering and complete withdrawal of corticosteroid and calcineurin inhibitors. | Relapse-free survival from complete drug withdrawal (relapse= presence of proteinuria or restart of corticosteroid or calcineurin inhibitors). | 48% of the children were in remission at 6 months. | No control. | No information. | No control. | 5 patients with bronchospasm, 3 patients with fever in combination with migrating skin rash and acute arthritis, 2 patients with neutropenia associated with transient viral infection and one patient with acute pyelonephritis with neutropenia. | No information. | No information. |
| **Source** | **Patients treated** | **RTX** | **Primary endpoint (1°) investigated** | **Secondary endpoint (2°) investigated** | **Verum reaching 1°** | **Control reaching 1°** | **Verum reaching 2°** | **Control reaching 2°** | **Adverse Events** | **QoL** | **Serious adverts events** |
| Magnasco et al., 2012 | 31 | 375mg/m^2^ at baseline and after 2 weeks. | Percentage change in daily proteinuria at 3 months. | No information. | Daily proteinuria: 0.32 (12% reduction, p=0.77). | Daily proteinuria: 0.36. | No information. | No information. | 1 patient with bronchospasm and hypotension, 1 patient with severe acute allergic reaction, 4 cases of abdominal pain, 3 cases of skin rash and 2 cases of mild dyspnoea. | No information. | No information. |
| Iijima et al., 2014 | 48 | 375mg/m^2^ weekly for 4 weeks | Relapse-free period | Time to treatment failure, relapse rate, time to four relapses of nephrotic syndrome, time to two relapses, time to transition to steroid resistance, steroid dose after randomisation, changes in steroid dose before and after randomisation, peripheral blood B cell count, peripheral blood B cell depletion period, human antichemieric antibody production rate, RTX blood concentration. | Age at disease onset and age at time of treatment did not affect the median relapse-free period in the RTX group. | No information. | Mean daily steroi dose: 9.12 mg/m^2^ (p<0.0001). | Mean daily steroid dose: 20.85mg/m^2^. | 24 patients (100%) had at least one AE. | No information. | 10 patients (42%) had at least one SAE. No deaths. |

| **Source** | **Patients treated** | **RTX** | **Primary endpoint (1°) investigated** | **Secondary endpoint (2°) investigated** | **Verum reaching 1°** | **Control reaching 1°** | **Verum reaching 2°** | **Control reaching 2°** | **Adverse Events** | **QoL** | **Serious adverts events** |
| --- | --- | --- | --- | --- | --- | --- | --- | --- | --- | --- | --- |
| Ravani et al., 2015 | 30 | 375mg/m^2^ once in combination with corticosteroid ≥0.7mg/kg/d for one month with consecutive corticosteroid tapering. Corticosteroid was restarted if proteinuria was ≥1g/m^2^/d. | Percentage change in daily proteinuria at 3 months. | Risk of relapse during the first year after treatment (relapse = proteinuria of 1000mg/m^2^/d associated with hypoalbuminemia). | No information. | No information. | Significant corticosteroid dose reduction at 3 months as compared to placebo (p<0.001). | No information. | All participants experienced nausea and/or skin rash during RTX infusions which resolved by slowing the infusion rate. One patient had fever with migrating skin rash and acute arthritis at the hip joint. | No information. | No information. |
| Basu et al., 2018 (RITURNS) | 120 | 375mg/m^2^ (maximum 500mg) weekly for 2-4 times. | Relapse-free survival rate at 12 months. | Frequency of relapses, time to first relapse, cumulative corticosteroid dosage, changes in serum biochemistry, peripheral blood B cell count, and number of children not receiving corticosteroids. | 90% of patients (p<0.001). | 63.3% of patients. | No information. | No information. | 41 patients with at least one AE. | No information. | No SAEs, no deaths. |
| **Neuromyelitis optica** | | | | | | | | | | | |
| **Source** | **Patients treated** | **RTX** | **Primary endpoint (1°) investigated** | **Secondary endpoint (2°) investigated** | **Verum reaching 1°** | **Control reaching 1°** | **Verum reaching 2°** | **Control reaching 2°** | **Adverse Events** | **QoL** | **Serious adverts events** |
| Nikoo et al., 2017 | 86 | 1000mg on week 0 and 2, repeated every 6 months. | Annual relapse rate measured after 12 months (relapse= new neurological symptoms or acute increase in EDSS). | EDSS after 12 months. | 78.8% of patients became relapse free (p=0.028). | 54.3% of patients became relapse free. | No information. | No information. | There was no significant difference in the occurrence of AEs between the two groups. | No information. | No clear information |

| **Pemphigus** | | | | | | | | | | | |
| --- | --- | --- | --- | --- | --- | --- | --- | --- | --- | --- | --- |
| **Source** | **Patients treated** | **RTX** | **Primary endpoint (1°) investigated** | **Secondary endpoint (2°) investigated** | **Verum reaching 1°** | **Control reaching 1°** | **Verum reaching 2°** | **Control reaching 2°** | **Adverse Events** | **QoL** | **Serious adverts events** |
| Kanwar et al., 2014 | 22 | 1000mg on days 0 and 15. | Time to complete consolidation phase, time to disease control, partial remission and complete remission (CR and PR as defined by the consensus statement of the International Pemphigus Committee). | Relapse rate, total cumulative dose of corticosteroids, adjuvant requirement and change in immunological markers. | No information. | No information. | 4 patients (36%) relapsed (p=0.2 in comparison to group B). | 7 patients (64%) relapsed. | Mean number of AE: 1.36. | No information. | No SAEs. |
| Joly et al., 2017 (Ritux 3) | 90 (74 patients with pemphigus vulgaris, 16 patients with pemphigus foliaceus). | 1000mg on days 1 and 14 and 500mg on months 12 and 18. Concomitant oral corticosteroid 0.5mg/kg/d for moderate pemphigus and 1.0mg/kg/d for severe pemphigus. After 1 month the dose was gradually reduced with the aim to stop corticosteroid after 3 months in patients with moderate pemphigus and after 6 months in patients with severe pemphigus. | Proportion of patients with complete remission off-therapy at month 24 (=absence of new or established lesions off corticosteroids for at least 2 months). | Delay to achievement of complete remission off-therapy, cumulative duration of complete remission off-therapy, relapse occurrence, cumulative dose of corticosteroid during the study, time change of dermatology quality of life index (DLQI) and Skindex QoL scores | 89% of patients (p<0.0001). | 34% of patients. | No information. | No information. | No information. | Significant better outcomes concerning the DLQI (p=0.0411) and Skindex score (p=0.0137). | No information on SAEs. No deaths. |

| **Rheumatoid arthritis** | | | | | | | | | | | |
| --- | --- | --- | --- | --- | --- | --- | --- | --- | --- | --- | --- |
| **Source** | **Patients treated** | **RTX** | **Primary endpoint (1°) investigated** | **Secondary endpoint (2°) investigated** | **Verum reaching 1°** | **Control reaching 1°** | **Verum reaching 2°** | **Control reaching 2°** | **Adverse Events** | **QoL** | **Serious adverts events** |
| Edwards et al., 2004 | 161 | 1000mg on days 1 and 15 in combination with placebos for cyclophosphamide and MTX. | Proportion of patients with an ACR50 response at week 24. | ACR20 and ACR70 response rates, change in disease-activity score, response according to EULAR response. | 33% of patients achieved primary endpoint (p=0.059). | 13% of patients achieved the primary endpoint. | ACR20 response rate: 65% (p≤0.025). ACR70 response rate: 15%. Significant improvement in the disease activity index: -2.2 (p≤0.002). Significantly higher proportion of patients responded to treatment according to the EULAR criteria 85% (p≤0.004). | ACR20 response rate: 38%. ACR70 response rate: 5%. No significant improvement in the disease activity index: -1.3. Lower proportion of patients responded to treatment according to the EULAR criteria 50%. | 36 patients (90%) with at least one AE during the first 48 weeks. | No information | 4 patients (10%) with at least one SAE during 48 weeks. 1 death due to fatal bronchopneumonia without causative organism. |
|  |  | 1000mg on days 1 and 15 in combination with CYC 750mg on days 3 and 17 plus placebo for MTX. | Proportion of patients with an ACR50 response at week 24. | ACR20 and ACR70 response rates, change in disease-activity score, response according to EULAR response. | 41% of patients achieved primary endpoint (p=0.005). |  | ACR20 response rate: 76%(p≤0.025). ACR70 response rate: 15%. Significant improvement in the disease activity index: -2.6 (p≤0.002). Significantly higher proportion of patients responded to treatment according to the EULAR criteria 85%(p≤0.004). |  | 35 patients (85%) with at least one AE during the first 48 weeks. | Statistically significant improvement in HAQ-DI in all treatment groups, as well as in the placebo group. | 7 patients (17%) with at least one SAE during 48 weeks. |
|  |  | 1000mg on days 1 and 15 in combination with MTX ≥10mg/week plus placebo for cyclophosphamide. | Proportion of patients with an ACR50 response at week 24. | ACR20 and ACR70 response rates, change in disease-activity score, response according to EULAR response. | 43% of patients achieved primary endpoint (p=0.005). |  | ACR20 response rate: 73%(p≤0.025). ACR70 response rate: 23% (p=0.048). Significant improvement in the disease activity index: -2.6 (p≤0.002). Significantly higher proportion of patients responded to treatment according to the EULAR criteria 83% (p≤0.004). |  | 35 patients (88%) with at least one AE during the first 48 weeks. | Statistically significant improvement in HAQ-DI in all treatment groups, as well as in the placebo group. | 4 patients (10%) with at least one SAE during 48 weeks. |
| Strand et al., 2006 (2 year follow-up of Edwards et al., 2004) | 128 | 1000mg on days 1 and 15. | No primary endpoint was defined for the follow-up period. | No secondary endpoint defined for the follow-up period. | ACR20 response rate: 8%, ACR50 response rate: 8%, ACR70 response rate: 3%, EULAR response rate: 16%. | ACR20 response rate: 13%, ACR50 response rate: 10%, ACR70 response rate: 8%, EULAR response rate: 16%. | No information. | No information. | No information. | No information on HAQ-DI at week 104. | No information. |
|  |  | 1000mg on days 1 and 15 in combination with CYC 750mg on days 3 and 17. | No primary endpoint was defined for the follow-up period. | No secondary endpoint defined for the follow-up period. | ACR20 response rate: 13%, ACR50 response rate: 10%, ACR70 response rate: 8%, EULAR response rate: 24%. |  | No information. |  | No information. | No information on HAQ-DI at week 104. | No information. |
|  |  | 1000mg on days 1 and 15 in combination with MTX ≥10mg/week. | No primary endpoint was defined for the follow-up period. | No secondary endpoint defined for the follow-up period. | ACR20 response rate: 33%, ACR50 response rate: 20%, ACR70 response rate: 10%, EULAR response rate: 39%. |  | No information. |  | No information. | No information on HAQ-DI at week 104. | No information. |
| Cohen et al., 2006 (REFLEX trial) | 499 | 1000mg on days 1 and 15. | Proportion of patients with an ACR20 response at week 24. | ACR50 and ACR70 response rates, DAS28, EULAR response criteria at 24 weeks. | 51% reached the primary endpoint (p<0.0001). | 18% reached the primary endpoint. | ACR50 response rate: 27% (p<0.0001). ACR70 response rate: 12% (p<0.0001). EULAR response of good or moderate: 65% (p<0.0001). | ACR50 response rate: 5%, ACR70 response rate: 1%. EULAR response of good or moderate: 22%. | 261 patients (85%) with at least one AE. | Significant reduction in HAQ DI at 24 weeks. Also fatigue was significantly reduced as measured by the FACIT-F. Furthermore significant improvements in SF-36 scores for mental and physical health occurred. | 23 patients (7%) with at least one SAE. No deaths. |

| **Source** | **Patients treated** | **RTX** | **Primary endpoint (1°) investigated** | **Secondary endpoint (2°) investigated** | **Verum reaching 1°** | **Control reaching 1°** | **Verum reaching 2°** | **Control reaching 2°** | **Adverse Events** | **QoL** | **Serious adverts events** |
| --- | --- | --- | --- | --- | --- | --- | --- | --- | --- | --- | --- |
| Keystone et al., 2009 (substudy of the REFLEX trial) | 463 | 1000mg on days 1 and 15. | Change in total Genant-modified Sharp score at week 56. | Erosion score, joint space narrowing score and proportion of patients with no further erosive damage. | No information. | No information. | Significantly more patients in the RTX groups experienced no erosive progression between baseline and week 56 (p=0.049). | See verum 2. | No information. | No information. | No information. |
| Emery et al., 2006 (DANCER trial) | 465 | 500mg on days 1 and 15 with no glucocorticoids, with i.v. premedication (100mg methylcorticosteroid) or with i.v. premedication plus oral corticosteroid 60mg on days 2-7 and 30mg on days 8 to 14. | Proportion of RF-positive patients with an ACR20 response at week 24. | ACR50 and ACR70 response rates, DAS28, EULAR response, fatigue, and HAQ-DI. | 55% of patients achieved an ACR20 response (p<0.0001). | 28% achieved an ACR 20 response. | ACR50 response: 33% (p≤ 0.001), ACR70 response: 13% (p=0.029). No statistical difference occurred between the RTX groups. Significant improvement in DAS28 (p<0.0001). Significantly more patients achieved an EULAR response of moderate or good (p<0.0001). | ACR50 response: 13%. ACR70 response: 5%. | 100 patients (81%) experienced at least one AE. | HAQ-DI improved from baseline to week 24: -0.43 as compared to 0.16 in the placebo group. Functional Assessment of Chronic Illness Therapy-Fatigue (FACIT-F) score improved by 20% as compared to 4% in the placebo group. | 9 SAEs (7%). 1 death due to cerebral infarction in a 73-year-old woman with known atrial fibrillation, hypertension, diabetes, and hyperlipidemia. |
|  |  | 1000mg on days 1 and 15 either with no glucocorticoids, with IV. premedication (100mg methylcorticosteroid) or with i.v. premedication plus oral corticosteroid 60mg on days 2-7 and 30mg on days 8 to 14. | Proportion of rheumatoid factor -positive patients with an ACR20 response at week 24. | ACR50 and ACR70 response rates, DAS28, EULAR response, fatigue, and HAQ-DI | 54% of patients achieved an ACR20 response (p<0.0001) |  | ACR50 response: 34% (p≤ 0.001), ACR70 response: 20% (p≤0.001). Significant improvement in DAS28 (p<0.0001). Significantly more patients achieved an EULAR response of moderate or good (p<0.0001). |  | 164 patients (85%) experienced at least one AE. | HAQ-DI improved from baseline to week 24: -0.49 as compared to -0.16 in the placebo group. FACIT-F score improved by 28% as compared to 4% in the placebo group. | 13 SAEs (7%). |
| **Source** | **Patients treated** | **RTX** | **Primary endpoint (1°) investigated** | **Secondary endpoint (2°) investigated** | **Verum reaching 1°** | **Control reaching 1°** | **Verum reaching 2°** | **Control reaching 2°** | **Adverse Events** | **QoL** | **Serious adverts events** |
| Owczarcyk et al., 2008 | 40 | 1000mg on days 1 and 15. | No primary endpoint defined. | No secondary endpoint defined. | No information. | No information. | No information. | No information. | No information. | No information. | No information. |
| Bingham et al., 2010 (SIERRA trial) | 100 | 1000mg on days 1 and 15 in combination with a stable dose of MTX (10-25mg/week). | Proportion of patients with a ≥4-fold rise in antitetanus IgG levels. | Measures of tetanus response and responses to pneumococcal vaccine, keyhole limpet hemocyanin, and C albicans skin test. | 39.1% reached primary endpoint. | 42.3% reached primary endpoint. | 77.4% of patients showed the same level of response in the delayed-type hypersensitivity response. | 70% of patients showed the same level of response in the delayed-type hypersensitivity response. | No information. | No information. | 3 patients experienced a SAE: hip fracture, chest pain, and coronary artery disease. No deaths. |
| Emery et al., 2010 (SERENE trial) | 509 | 500mg on days 1 and 15. If not in remission at week 24, another treatment course was applied. | Proportion of patients with an ACR20 response at week 24. | Proportion of patients with an ACR50/70 response, EULAR responses, DAS28-ESR, Health Assessment Questionnaire Disability Index (HAQ-DI), SF-36, and FACIT-F. | 54.5% (p<0.0001) | 23.3% | ACR50 response rate: 26.3% (p<0.0001). The proportion of patients achieving an ACR70 response was greater than in the placebo group but the difference was not statistically significant. | ACR50 response rate: 9.3%. The proportion of patients achieving an ACR70 response was greater than in the placebo group but the difference was not statistically significant. | 128 patients (77%) experienced at least one AE. | Statistically significant improvements in HAQ-DI, FACIT-F, and SF-36 scores. | 6 patients (4%) experienced at least one SAE. 3 deaths: interstitial lung disease and abdominal sepsis and unknown cause. |
|  |  | 1000mg on days 1 and 15. If not in remission at week 24, another treatment course was applied. | Proportion of patients with an ACR20 response at week 24. | Proportion of patients with an ACR50/70 response, EULAR responses, DAS28-ESR, HAQ-DI, SF-36, and FACIT-F. | 50.6% (p<0.0001). |  | ACR50 response rate: 25.9% (p<0.0001). The proportion of patients achieving an ACR70 response was greater than in the placebo group but the difference was not statistically significant. |  | 130 patients (76%) experienced at least one AE. | Statistically significant improvements in HAQ-DI, FACIT-F, and SF-36 scores. | 15 patients (9%) experienced at least one SAE. One death (unknown cause). |

| **Source** | **Patients treated** | | **RTX** | | **Primary endpoint (1°) investigated** | | **Secondary endpoint (2°) investigated** | | **Verum reaching 1°** | | **Control reaching 1°** | | **Verum reaching 2°** | | **Control reaching 2°** | | **Adverse Events** | | **QoL** | | **Serious adverts events** | |
| --- | --- | --- | --- | --- | --- | --- | --- | --- | --- | --- | --- | --- | --- | --- | --- | --- | --- | --- | --- | --- | --- | --- |
| Mease et al., 2010 (SUNRISE trial) | 475 (559 received the open-label first course of RTX) | | 1000mg on days 1 and 15 at week 24. | | Proportion of patients with an ACR20 response at week 48. | | ACR50/70 response rates, change from baseline to week 48 in DAS28-ESR, proportion of patients with ELAR responses. | | 54% (p=0.0195). | | 45.0%. | | Patients who did not respond to the first course were no more likely to respond to a second course of RTX than to placebo. | |  | | 226 patients (71%) with at least one AE. | | No difference in HAQ-DI score. | | 22 patients (7%) with at least one SAE. 2 deaths: acute respiratory distress syndrome and cardiorespiratory arrest. | |
| Rubbert-Roth et al., 2010 (MIRROR trial) | 377 | | 500mg on days 1 and 15 with a repeated course of again 2x500mg at week 24. | | Proportion of patients with an ACR20 response at week 48. | | ACR50/70 response rates, change from baseline to week 48 in DAS28-ESR, EULAR response, change from baseline in SF-36 and FACIT-F. | | 64% (no significant difference between dose groups). | | No control. | | Moderate or good EULAR response: 73%. | | No control. | | 121 patients (90%) experienced at least one AE. | | HAQ-DI, SF-36 and FACIT-F improved in all dosing groups with no significant in-between difference. | | 15 patients (11%) experienced at least one SAE. No deaths. | |
|  |  |  | 500mg on days 1 and 15 with a repeated course of 2x1000mg at week 24. | | Proportion of patients with an ACR20 response at week 48. | | ACR50/70 response rates, change from baseline to week 48 in DAS28-ESR, EULAR response, change from baseline in SF-36 and FACIT-F. | | 64% (no significant difference between dose groups). | | No control. | | Moderate or good EULAR response: 72%. | | No control. | | 106 patients (89%) experienced at least one AE. | | HAQ-DI, SF-36 and FACIT-F improved in all dosing groups with no significant in-between difference. | | 21 patients (18%) experienced at least one SAE. No deaths. | |
|  |  |  | 1000mg on days 1 and 15 with a repeated course of again 2x1000mg at week 24. | | Proportion of patients with an ACR20 response at week 48. | | ACR50/70 response rates, change from baseline to week 48 in DAS28-ESR, EULAR response, change from baseline in SF-36 and FACIT-F. | | 72% (no significant difference between dose groups). | | No control. | | Moderate or good EULAR response: 89%. EULAR responses were achieved by significantly more patients in this group compared to other dosing groups (p=0.0495). | | No control. | | 85 patients (91%) experienced at least one AE. | | HAQ-DI, SF-36 and FACIT-F improved in all dosing groups with no significant in-between difference. | | 16 patients (17%) experienced at least one SAE. No deaths. | |
| **Source** | **Patients treated** | | **RTX** | | **Primary endpoint (1°) investigated** | | **Secondary endpoint (2°) investigated** | | **Verum reaching 1°** | | **Control reaching 1°** | | **Verum reaching 2°** | | **Control reaching 2°** | | **Adverse Events** | | **QoL** | | **Serious adverts events** | |
| Greenwald et al., 2011 (TAME trial) | 51 | | 500mg on days 1 and 15 | | Proportion of patients with at least 1 serious infection at week 24. | | ACR20/50/70 response rates at week 24 | | 3%: 1 patient developed pneumonia 45 days after the second RTX infusion, which resolved after adequate treatment. | | 0.0%. | | 5 patients (15%) experienced a grade 3 AE. 55% of patients developed infections. The most common infections were upper respiratory tract infections and sinusitis. Mean duration was 10.7 days. | | No patient experienced a grade 3 AE. 61% of patients developed infections. The most common infections were upper respiratory tract infections and sinusitis. Mean duration was 10.8 days. | | 31 patients (94%) experienced at least one AE. | | HAQ-DI improved more in RTX treated patients but the study was not powered for statistical analysis. | | 2 patients (6%) with at least one SAE: pneumonia and coronary artery occlusion (history of 4-vessel coronary artery bypass surgery). No deaths. | |
| Tak et al., 2011 (IMAGE trial) | 748 | | 500mg on days 1 and 15. Retreatment was permitted after week 24 if DAS28 was ≥2.6. | | Change in total Genant-modified sharp score (mTSS) from baseline to week 52. | | No clear definition of secondary endpoints. | | No information. | | No information. | | No information. | | No information. | | 189 patients (76%) experienced at least one AE. | | HAQ-DI significantly improved at week 52 as compared to placebo. | | 23 patients (9%) experienced at least one SAE. No deaths. | |
|  |  |  | 1000mg on days 1 and 15. Retreatment was permitted after week 24 if DAS28 was ≥2.6. | | Change in total mTSS from baseline to week 52. | | No clear definition of secondary endpoints. | | No information. | |  |  | No information. | |  |  | 197 patients (79%) experienced at least one AE. | | HAQ-DI significantly improved at week 52 as compared to placebo. | | 24 patients (10%) experienced at least one SAE. No deaths. | |
| Tak et al., 2012 (Extension of the IMAGE trial) | 748 (606 completed 104 weeks) | | 500mg on days 1 and 15. Retreatment was permitted after week 24 if DAS28 was ≥2.6. | | No primary endpoint defined. | | Change in mTSS, total erosion score and joint space narrowing score from baseline to week 104. | | No information. | | No information. | | Main progressive joint damage occurred from baseline to week 24. Thereafter only minimal change occurred. | | Progressive joint damage develops in a near-linear fashion. | | 206 patients (83%) with at least one AE. | | HAQ-DI significantly improved at week 104 as compared to placebo. | | 37 patients (15%) with at least one SAE. 2 deaths: pneumonia and unknown reason. | |
|  |  |  | 1000mg on days 1 and 15. Retreatment was permitted after week 24 if DAS28 was ≥2.6. | | No primary endpoint defined. | | Change in mTSS, total erosion score and joint space narrowing score from baseline to week 104. | | No information. | |  |  | Main progressive joint damage occurred from baseline to week 24. Thereafter only minimal change occurred. | |  |  | 217 patients (87%) with at least one AE. | | HAQ-DI significantly improved at week 104 as compared to placebo. | | 33 patients (13%) with at least one SAE. 1 death: duodenal cancer. | |
| **Source** | **Patients treated** | | **RTX** | | **Primary endpoint (1°) investigated** | | **Secondary endpoint (2°) investigated** | | **Verum reaching 1°** | | **Control reaching 1°** | | **Verum reaching 2°** | | **Control reaching 2°** | | **Adverse Events** | | **QoL** | | **Serious adverts events** | |
| Mariette et al., 2014 (SMART trial) | 143 | | 1000mg on day 1 of week 24. Further retreatments were scheduled based on DAS28 >3.2. | | DAS28-CRP area under the curve over 104 weeks with a non-inferiority margin defined by 20% of the mean DAS28-CRP of the reference data. | | DAS28-CRP, ACR20/50/70, EULAR response, low disease activity, remission rates, tender joint count, swollen joint count, number of required RTX courses, time to second retreatment, safety. | | No information. | | No information. | | No information. | | No information. | | 61 patients (92%) experienced at least one treatment AE. | | No significant difference in QoL was observed between the two treatment arms. | | 19 patients (29%) experienced at least one SAE. | |
| Vital et al., 2015 | 25 | | 1000mg at week 2 and one infusion of placebo at week 4. Further RTX courses (2x1000mg) were allowed after week 28 if DAS increased at least 0.6 points. | | Proportion of patients achieving a ACR20 response at week 28. | | EULAR and ACR20/50/70 response at weeks 14, 28, 40, and 52, numbers of B cells, immunoglobulin titres, and adverse event rates | | 62.0% | | 67.0% | | Week 40: ACR20 response: 31%. EULAR good or moderate response: 54%. WEEK 52: ACR20 response: 8%. EULAR good or moderate response: 17%. Differences in ACR50/70 and EULAR good response rates as well as immunoglobulin titres were not significant. | | Week 40: ACR20 response: 75% (p=0.027). EULAR good or moderate response: 92% (p=0.035). WEEK 52: ACR20 response: 45% (p=0.043). EULAR good or moderate response: 64% (p=0.021). Differences in ACR50/70 and EULAR good response rates as well as immunoglobulin titres were not significant. | | 54 AEs occurred during 12 months | | No information. | | 1 SAE: elective surgery hospitalization. | |
| Peterfy et al., 2016 (RA-SCORE trial) | 185 | | 500mg on days 1 and 15. Retreatment was permitted after week 24 if DAS28 was ≥2.6. | | Change in RAMRIS erosion score from baseline to week 24 | | Change from baseline in RAMRIS erosion score at week 12 and 52, RAMRIS synovitis and osteitis at weeks 12, 24, and 52, and porportion of patients with no progression in RAMRIS erosion score at weeks 24, and 52. | | No information. | | No information. | | No information. | | No information. | | 35 patients (56.5%) experienced at least one TEAE | | No information. | | 3 patients (4.8%) experienced at least one SAE. No deaths | |
|  |  |  | 1000mg on days 1 and 15. Retreatment was permitted after week 24 if DAS28 was ≥2.6. | | Change in RAMRIS erosion score from baseline to week 24 | | Change from baseline in RAMRIS erosion score at week 12 and 52, RAMRIS synovitis and osteitis at weeks 12, 24, and 52, and porportion of patients with no progression in RAMRIS erosion score at weeks 24, and 52. | | No information. | |  |  | No information. | |  |  | 36 patients (60%) experienced at least one TEAE | | No information. | | 4 patients (6.7%) experienced at least one SAE. No deaths. | |
| **Sjögren’s syndrome** | | | | | | | | | | | | | | | | | | | | | | |
| **Source** | | **Patients treated** | | **RTX** | | **Primary endpoint (1°) investigated** | | **Secondary endpoint (2°) investigated** | | **Verum reaching 1°** | | **Control reaching 1°** | | **Verum reaching 2°** | | **Control reaching 2°** | | **Adverse Events** | | **QoL** | | **Serious adverts events** |
| Dass et al., 2008 | | 17 | | 1000mg on days 1 and 15 | | 20% improvement on fatigue visual analogue scale (VAS) score at 6 months | | SF-36 questionnaire, Functional Assessment of Chronic Illness Therapy-Fatigue, Profile of Fatigue and Discomfort questionnaire, sicca score, combined fatigue and discomfort score, Schirmer-1 test, ESR, and CRP. | | 87.5% (p=0.36). | | 55.6% | | Mean improvement in fatigue VAS at 6 months: 49.5% (p=0.24). | | Mean improvement in fatigue VAS at 6 months: 20.5%. | | No clear information. 2 patients experienced infusion reactions. | | Significant improvement in social functioning score of SF-36 after 6 months of RTX therapy (p=0.01). Difference in mental health domain score of SF-36 was not significant (p=0.06). No significant change was seen in the mental component summary of the SF-36, the physical health component of SF-36 and pain VAS. Significant reduction in Profile of Fatigue and Discomfort questionnaire (p=0.026). | | 3 SAEs were observed in 2 patients: serum sickness in 1 patient, gastroenteritis and palpitations leading to 2 hospitalizations in another patient. No information on deaths. |
| Meijer et al., 2010 | | 30 | | 1000mg on days 1 and 15 | | Significant improvement in stimulated whole saliva flow rate. | | Salivary/lacrimal function, immunologic variables, subjective variables. | | No information. | | No information. | | Complete B cell depletion was achieved. | | No significant change in B cell number. | | No clear information. | | Significant improvement in MFI score and SF-36 score for vitality as compared with placebo was achieved. Furthermore all VAS scores for oral and ocular sicca symptoms improved significantly. | | No information. |
| Devauchelle-Pensec et al., 2014 (TEARS trial) | | 120 | | 1000mg on days 1 and 15. | | Improvement of at least 30mm in 2 of 4 VASs by week 24. | | Variations from baseline in the individual VAS scores at weeks 6 and 16, disease activity, systemic manifestations and treatment activity as assessed by the investigator. | | 23.0% of patients achieved the primary endpoint. This was not a significant difference to patients treated with placebo. | | 22% of patients achieved the primary endpoint. | | 37.5% of patients achieved a 30-mm decrease in the VAS fatigue score at week 6 (p<0.001) and 32.6% at week 16 (p=0.012). At week 24 significant improvements were seen in the levels of IgG, IgA, IgM, and beta2-microglobulin. BAFF did not decrease significantly. | | 15.7% of patients achieved a 30-mm decrease in the VAS fatigue score at week 6 and 4.1% at week 16. | | 55 patients experienced at least one AE (87.3%). Infusion reactions occurred significantly more often in the RTX group. | | No information. | | 13 patients (20.6%) experienced at least one SAE. No deaths. |
| Bowman et al., 2017 (TRACTISS) | | 131 | | 1000mg at weeks 0, 2,24 and 26 | | Achievement of a reduction of at least 30% in the VAS assessment of either fatigue or oral dryness at week 48. | | Measurements of salivary and lacrimal flow | | 39.3% (p=0.76) | | 0.375 | | No information. | | No information. | | 61 patients with at least one AE | | No difference concerning SF-36 | | 9 patients with at least one SAE. No deaths |
| Fisher et al., 2018 (substudy of TRACTISS) | | 52 | | 1000mg at weeks 0, 2,24 and 27 | | Total ultrasound score at weeks 16 and 48 as compared to baseline | | No secondary endpoints defined | | No information. | | No information. | | No information. | | No information. | | See original study | | See original study | | See original study |
| **Spondyloarthropathy** | | | | | | | | | | | | | | | | | | | | | | |
| **Source** | | **Patients treated** | | **RTX** | | **Primary endpoint (1°) investigated** | | **Secondary endpoint (2°) investigated** | | **Verum reaching 1°** | | **Control reaching 1°** | | **Verum reaching 2°** | | **Control reaching 2°** | | **Adverse Events** | | **QoL** | | **Serious adverts events** |
| Song et al., 2010 | | 20 (10 TNF inhibitor naive and 10 TNF inhibitor failures) | | 1000mg twice given 2 weeks apart. Additional courses were given upon flare. | | Proportion of patients with ASAS20 response at week 24. | | Proportion of patients with a ASAS40 response, a BASDAI20 response, or a BASDAI50 response, partial remission according to ASAS criteria, and mean improvement in BASDAI, BASFI and health-related QoL. | | 40% of patients achieved ASAS20 response. | | No control. | | Combined response rates: 25% of patients achieved ASAS40 response, 15% achieved partial remission, 40% achieved BASDAI20 repsonse and 25% achiev BASDAI50 response. Patients naive to TNF inhibitors showed higher response rates according to ASAS20 (50%), ASAS40 (40%), partial remission (30%), BASDAI20 (60%), and BASDAI50 (50%). | | No control. | | 90 AEs occurred until week 24. The most common were upper respiratory tract infections and infusion reactions. | | No clear information | | 5 SAEs: surgical treatment of haemorrhoids prior to baseline, ulnar fracture, nephrolithiasis, skin abscess, and gastrointestinal bleeding. None of these were considered as related to study drug. No information on deaths. |
| Song et al., 2013 (Follow-up trial of Song et al., 2010) | | 9 | | 1000mg twice given 2 weeks apart. Additional courses were given upon flare. | | No primary endpoint defined | | No secondary endpoint defined | | No information. | | No control. | | No information. | | No control. | | No information. | | No clear information | | No information. |
| Jimenez et al., 2012 | | 9 | | 1000mg on days 0 and 14 | | Psoriatic arthritis response criteria (PsARC) improvement by 30% of tender and swollen joint count or if only one fulfilled, an additional 30% improvement of VAS patient global assessment or evaluator global assessment. | | Changes in DAS28, simplified disease activity index, clinical disease activity index, disease activity index for PsA, and HAQ. | | 56% of patients achieved ≥30% improvement by PsARC. | | No control. | | No information. | | No control. | | No clear information | | HAQ improved significantly (p=0.03) | | No SAEs. No information on deaths. |

| **Systemic lupus erythematosus** | | | | | | | | | | | |
| --- | --- | --- | --- | --- | --- | --- | --- | --- | --- | --- | --- |
| **Source** | **Patients treated** | **RTX** | **Primary endpoint (1°) investigated** | **Secondary endpoint (2°) investigated** | **Verum reaching 1°** | **Control reaching 1°** | **Verum reaching 2°** | **Control reaching 2°** | **Adverse Events** | **QoL** | **Serious adverts events** |
| Andrade-Ortega et al., 2010 | 19 | 1000mg on days 1 and 15. | No information. | No information. | Remission (MEX-SLEDAI<3) was achieved in 8 patients after 6 months and in 9 patients after a year. | Remission (MEX-SLEDAI<3) was achieved in 6 patients after 6 months and in 8 patients after 1 year. | No significant differences in the cumulative corticosteroid dosis were observed. | No significant differences in the cumulative corticosteroid doses were observed. | Both medications were well tolerated. | No information. | 2 SAE: septic arthritis and hemarthrosis. |
| Merrill et al., 2010 (EXPLORER trial) | 257 | 1000mg on days 1, 15, 168, and 182. | Achieving and maintaining a major clinical response, a partial clinical response, or no clinical response at week 52 (Major clinical response = BILAG C scores or better in all organs at 24 weeks without experiencing a severe flare, and maintaining this response without a moderate or severe flare to week 52. Partial response = achieving BILAG C scores or better at week 24 and maintaining this response without a new BILAG A or B score for 16 consecutive weeks / achieving no more than 1 organ with a BILAG B score at week 24 without achieving one new BILAG A or B score to week 52 / achieving a maximum of 2 BILAG B scores at week 24 without developing BILAG A or B scores in dew domains until week 52 if baseline BILAG score for the patient was 1A score plus ≥2B scores, ≥2A scores, or ≥4 B scores. No clinical response: failure to meet the definition of major or partial clinical response). | Proportion of patients with a major clinical response, proportion of patients with a partial clinical response, proportion of patients with a BILAG C score or better in all organs at week 24, time to first moderate or severe flare, improvement in QoL as measured by the lupus QoL, proportion of patients with a major clinical response and a corticosteroid dose of <10mg/d from week 24 to 52. | 12.4% had a major clinical response, 17.2% had a partial clinical response (no significant difference). However African American/Hispanic patients achieved significantly better major and partial clinical responses with RTX than placebo (p=0.0408) | 15.9% had a major clinical response, 12% had a partial clinical response. | Proportion of patients with a BILAG C score or better after 24 weeks: 24.9% | Proportion of patients with a BILAG C score or better after 24 weeks: 27.3% | RTX patients experienced more grade 3 and grade 4 neutropenias. The number of infectious AEs was comparable. The proportion of patient with serious infections was greater in the placebo group. | No significant difference in the change of SF-36 QoL physical component summary score. | 64 patients (37.9%) had at least one SAE. Only 13 were considered as treatment related. 4 deaths (perforated bowel, multiple drug intoxication, unknown cause, and neonatal death). |
| Rovin et al., 2012 (LUNAR trial) | 144 | 1000mg on days 1, 15, 168, and 182 | Renal response status at week 52 (CRR= normal serum creatinine level if it was abnormal at baseline, or serum creatinine level of ≤115% of baseline if it was normal at baseline, inactive urinary sediment, and UPC ratio <0.5, PRR= serum creatinine level ≤115% of baseline, RBCs/hpf ≤50% above baseline and no RBC casts, and at least a 50% decrease in the UPC ratio to <1.0, NR= CRR and PRR criteria not met) | CRR sustained from week 24 to week 52, CRR rates at week 52, reduction in the baseline UPC ratio from >3.0 to <1.0 at week 52, time to first CRR, time-adjusted AUC minus the baseline BILAG score over 52 weeks, change in SF-36. | CRR: 26.4% of patients, PRR: 30.6% of patients, NR: 43.1% of patients (p=0.55). Overall renal response rate: 56.9% (p=0.18). Black patients had higher renal response rates when treated with RTX (70%) as compared to placebo (45%). | CRR: 30.6% of patients, PRR: 15.3% of patients, NR: 54.2% of patients. Overall renal response rate: 45.8% | No information. | No information. | 72 patients (98.6%) experienced at least one AE | No significant difference in the change of SF-36 QoL physical function score. | 24 patients (32.9%) had at least one AE. 2 deaths (both considered as unrelated to RTX): sepsis secondary to S. aureus infection and alveolar hemorrhage. |
| Zhang et al., 2015 | 84 | 375mg/m^2^ at weeks 0, 2, 4, and 6 in combination with 800mg CYC at weeks 1 and 3 | No primary endpoint | No secondary endpoints | 64.3% with CR, 19% with PR, 16.7% with NR | 21.4% with CR; 35.7% with PR, 42.9% with NR | No information. | No information. | No information. | No information. | No information. |
| **Systemic sclerosis** | | | | | | | | | | | |
| **Source** | **Patients treated** | **RTX** | **Primary endpoint (1°) investigated** | **Secondary endpoint (2°) investigated** | **Verum reaching 1°** | **Control reaching 1°** | **Verum reaching 2°** | **Control reaching 2°** | **Adverse Events** | **QoL** | **Serious adverts events** |
| Daoussis et al., 2010 | 14 | 375mg/m2 weekly for 4 weeks with a repeated course after 6 months. | Change in pulmonary function as assessed by pulmonary function test and clinical assessment of skin involvement by the MRSS (modified Rodnan skin score). | Change in skin histology, HRCT scores, serum levels of soluble markers, and overall functional impairment. | No information | No information | No information | No information | Patient 4 suffered from a respiratory tract infection and was hospitalized for 3 days. | Significant improvement in HAQ scores after 1 year as compared to control group | No information |
| **Ulcerative colitis** | | | | | | | | | | | |
| **Source** | **Patients treated** | **RTX** | **Primary endpoint (1°) investigated** | **Secondary endpoint (2°) investigated** | **Verum reaching 1°** | **Control reaching 1°** | **Verum reaching 2°** | **Control reaching 2°** | **Adverse Events** | **QoL** | **Serious adverts events** |
| Leiper et al., 2011 | 24 | 1000mg on weeks 0 and 2 | Remission at 4 weeks (=Mayo score ≤2) | Clinical response (=Mayo score reduction of at least 3 points) at weeks 4 and 12, remission at weeks 8 and 12, endoscopic mucosal healing at weeks 4 and 12, and improvement in the IBD-specific QoL Index at weeks 4 and 12. | 19% achieved remission (p=1.0) | 12.5% achieved remission. | 50% of the patients achieved clinical response at week 4, but the difference in comparison to placebo was lost at week 8. No significant difference in reduction of Mayo score occurred at week 8 or 12. | 25% of the patients achieved clinical response at week 4. No significant difference in reduction of Mayo score occurred at week 8 or 12. | 15/16 patients experienced AEs | No significant improvement in IBDQ was observed. | 3 SAE: flare up of UC, pulmonary embolism, pregnancy. No information on deaths. |
